# Moving toward generalizable NZ-1 labeling for 3D structure determination with optimized epitope-tag insertion

**DOI:** 10.1107/S2059798321002527

**Published:** 2021-04-19

**Authors:** Risako Tamura-Sakaguchi, Rie Aruga, Mika Hirose, Toru Ekimoto, Takuya Miyake, Yohei Hizukuri, Rika Oi, Mika K. Kaneko, Yukinari Kato, Yoshinori Akiyama, Mitsunori Ikeguchi, Kenji Iwasaki, Terukazu Nogi

**Affiliations:** a Graduate School of Medical Life Science, Yokohama City University, Japan; b Institute for Protein Research, Osaka University, Japan; c Institute for Frontier Life and Medical Sciences, Kyoto University, Japan; d Department of Antibody Drug Development, Tohoku University Graduate School of Medicine, Japan; e New Industry Creation Hatchery Center, Tohoku University, Japan; f RIKEN, Medical Life Sciences Innovation Hub Program, Japan; g Life Science Center for Survival Dynamics, Tsukuba Advanced Research Alliance (TARA), University of Tsukuba, Japan

**Keywords:** antibody labeling, epitope insertion, antibody-assisted structural analysis, electron microscopy, protein crystallography

## Abstract

Antibody labeling has been conducted extensively for structure determination using both X-ray crystallography and electron microscopy (EM). However, establishing target-specific antibodies is a prerequisite for applying antibody-assisted structural analysis. To expand the applicability of this strategy, an alternative method has been developed to prepare an antibody complex by inserting an exogenous epitope into the target. It has already been demonstrated that the Fab of the NZ-1 monoclonal antibody can form a stable complex with a target containing a PA12 tag as an inserted epitope. Nevertheless, it was also found that complex formation through the inserted PA12 tag inevitably caused structural changes around the insertion site on the target. Here, an attempt was made to improve the tag-insertion method, and it was consequently discovered that an alternate tag (PA14) could replace various loops on the target without inducing large structural changes. Crystallographic analysis demonstrated that the inserted PA14 tag adopts a loop-like conformation with closed ends in the antigen-binding pocket of the NZ-1 Fab. Due to proximity of the termini in the bound conformation, the more optimal PA14 tag had only a minor impact on the target structure. In fact, the PA14 tag could also be inserted into a sterically hindered loop for labeling. Molecular-dynamics simulations also showed a rigid structure for the target regardless of PA14 insertion and complex formation with the NZ-1 Fab. Using this improved labeling technique, negative-stain EM was performed on a bacterial site-2 protease, which enabled an approximation of the domain arrangement based on the docking mode of the NZ-1 Fab.

## Abstract 2

An improved antibody-labeling method using an inserted exogenous epitope is presented, in which binding of the antibody has less impact on the folding of the target.

## 1. Introduction   

Antibody labeling has become a useful tool for determining the structures of protein molecules and complexes. It has been established that the antigen-binding fragment (Fab) and the variable fragment (Fv) can bind to their target and serve as chaperones to promote crystallization in X-ray crystallo­graphy, as discussed in greater depth in many previous reviews (Hino *et al.*, 2013 ▸; Hunte & Michel, 2002 ▸; Koide, 2009 ▸). Accordingly, a large number of crystal structures have successfully been determined using antibody fragments as crystallization chaperones. Antibody labeling has been partic­ularly useful for determining membrane-protein structures. The first use of antibody labeling to facilitate the crystallization of a membrane protein was in the structural analysis of bacterial cytochrome *c* oxidase in complex with an Fv fragment (Ostermeier *et al.*, 1995 ▸). Subsequently, a high-resolution crystal structure of the KcsA K^+^ channel was successfully determined using a Fab as a crystallization chaperone (Zhou *et al.*, 2001 ▸). Utilizing Fabs has also accelerated the structure determination of G-protein-coupled receptors such as the human β_2_ adrenergic receptor (Rasmussen *et al.*, 2007 ▸; Day *et al.*, 2007 ▸). As a consequence, the usefulness of antibody labeling has been broadly accepted in structural biology. Nevertheless, establishing antibodies that stably bind to their respective targets is a prerequisite for utilizing antibody labeling that limits its applicability.

**Figure 1:**
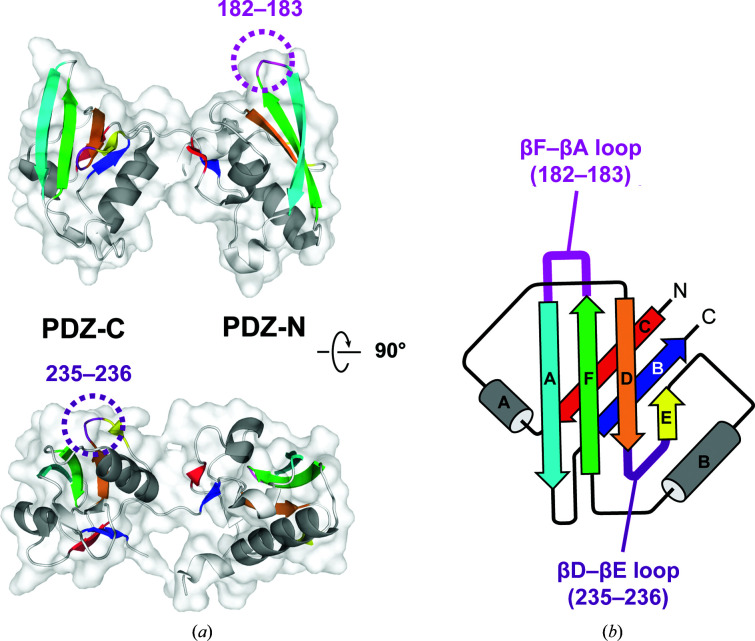
Design of PA14-insertion sites in the PDZ tandem of *Aa*RseP. (*a*) 3D structure of the PDZ tandem of *Aa*RseP. The PDZ tandem structure (PDB entry 3wkm, chain *A*) is shown as a ribbon model with a transparent surface in two different views. Each PDZ domain is colored by its six β-strands. The α-­helices conserved in the PDZ fold are colored gray. The two PA14-insertion sites are highlighted with dotted circles. (*b*) Topology diagram. Both of the PDZ domains in *Aa*RseP are circular permutants of the canonical PDZ fold. The first mutant was constructed by replacing residues 182–183 of the βF–βA loop of the PDZ-N domain with the PA14 tag. In the second mutant, PA14 was inserted between residues 235 and 236 of the βD–βE loop of the PDZ-C domain.

To make antibody-assisted structural analysis more immediately applicable, we developed an alternative strategy in which antibody labeling is mediated through an exogenous epitope sequence that is inserted into the target. Specifically, we utilized the PA tag–NZ-1 antibody pair. The high-affinity NZ-1 monoclonal antibody was established by immunizing rats with a tetradecapeptide (EGGVAMPGAEDDVV) from the platelet-aggregation-stimulating domain of human podoplanin (Kato *et al.*, 2006 ▸). It was also shown that a truncated dodecapeptide (GVAMPGAEDDVV) can bind to NZ-1 with an affinity comparable to that of the original epitope (Fujii *et al.*, 2014 ▸), and this has been developed as a PA tag for affinity purification and specific labeling (in this study, for clarity, the PA tag is referred to as the PA12 tag, while the original epitope composed of 14 residues is referred to as the PA14 tag). The mode of recognition was examined by determining the crystal structure of the NZ-1 Fab in complex with the PA14 peptide (Fujii *et al.*, 2016 ▸). The structure shows that the PA12 portion of the PA14 peptide binds stably to NZ-1 and adopts a bent loop-like conformation in the antigen-binding pocket of NZ-1 (Supplementary Fig. S1*a*). These observations prompted the prediction that the PA12 tag could bind to NZ-1 either when fused to the termini of the target or when inserted into a loop region. In fact, our previous study demonstrated the preparation of multiple crystallizable complexes with the PA12 tag inserted into loop regions protruding from a globular domain of the target (Tamura *et al.*, 2019 ▸). Our target protein was an integral membrane protein from the hyperthermophile *Aquifex aeolicus* (Deckert *et al.*, 1998 ▸). This membrane protein is an orthologue of the *Escherichia coli* intramembrane protease RseP that belongs to the site-2 protease family (Hizukuri *et al.*, 2017 ▸; hereafter, *E. coli* RseP and the *A. aeolicus* orthologue are referred to as *Ec*RseP and *Aa*RseP, respectively). Like *Ec*RseP, *Aa*RseP possesses two tandemly arranged PSD95/Dlg/ZO-1 (PDZ) domains in the periplasmic soluble region, referred to as the PDZ tandem (Fig. 1 ▸ *a*). The two PDZ domains in the tandem are each composed of six β-strands and two α-helices (Hizukuri *et al.*, 2014 ▸). Both of the PDZ domains are circular permutants of the canonical PDZ fold (Fig. 1 ▸ *b*). In the PDZ domains of *Ec*RseP and *Aa*RseP, the new termini are formed by a chain break between strands βB and βC of the canonical fold, and the canonical termini on the opposite side of the PDZ domain at strands βA and βF are connected by a hairpin loop. We have demonstrated that the PA12 tag can be inserted into the βF–βA loops of the two PDZ domains without disrupting their structures and that the PA12-inserted PDZ tandem fragment formed a complex with the NZ-1 Fab (Tamura *et al.*, 2019 ▸).

Our previous study also revealed that more rigid complexes could be prepared by adjusting the insertion point to reduce the number of residues that undergo structural change upon binding to the NZ-1 Fab (Tamura *et al.*, 2019 ▸). However, such residues could not be eliminated completely. For instance, the inter-strand hydrogen bonds of the βF–βA loops were broken around the junctions because the C^α^ atoms of both ends of the PA12 tag were separated by 12–14 Å when accommodated in the antigen-binding pocket of NZ-1. This separation seemed to be inevitable as long as we utilized the PA12 tag as the inserted epitope. Therefore, in this study we tested whether the structural change in the target could be reduced by modifying the tag sequence. Specifically, we utilized the PA14 tag, instead of PA12, which contains two additional residues (Glu-Gly) upstream of PA12. These two residues were flexible in the co-crystal structure of the NZ-1 Fab with the PA14 peptide, and they may be able to change conformation to accommodate the fold of the target protein near the insertion site. Here we again utilized the PDZ tandem of *Aa*RseP as the target for PA14 insertion and complex formation with the NZ-1 Fab. In addition to structure determination by X-ray crystallography, we examined the dynamic properties of the PA14-mediated Fab–PDZ complexes through all-atom molecular-dynamics simulations. Finally, we approximated the spatial arrangement of the two PDZ domains in PA14-inserted full-length *Aa*RseP using negative-stain electron microscopy (EM) together with our improved NZ-1 labeling technique.

## 2. Materials and methods   

### 2.1. Plasmid construction for expression and *in vivo* cleavage assay   

The pGEX-2T-based plasmid for the PDZ tandem fragment (residues 115–292), which was constructed in our previous study (Hizukuri *et al.*, 2014 ▸), is hereafter termed pNO1499. The expression plasmids for the PA14-inserted PDZ tandem mutants were constructed by inverse PCR on pNO1499 using primers containing the respective mutations. The PCR products were transformed into *E. coli* XL-1 Blue cells after digestion of the pNO1499 template with DpnI. The resultant plasmids for the PDZ tandem (181-PA24-184) and (235-PA14-236) mutants are pNY1493 and pNY1468, respectively.

The DNA encoding full-length *Aa*RseP fused with a C-terminal tag, Gly-Arg-Gly-Ser-His_8_ (*Aa*RseP-His_8_), was amplified by PCR using the genomic DNA of *A. aeolicus* strain VF5 and primers encoding the C-terminal tag sequence. The amplified DNA was first cloned into the NdeI/BamHI sites of the pET-11c plasmid. Subsequently, the DNA encoding *Aa*RseP-His_8_ together with the Shine–Dalgarno sequence was extracted and cloned into the EcoRI/BamHI sites of the pUC118 plasmid, resulting in the plasmid pNO1457. The expression plasmids for the *Aa*RseP mutants, pNO1461 (active-site mutant E18Q), pNY1493 (181-PA14-184) and pNY1478 (235-PA14-236), were constructed by introducing the respective mutations into pNO1457 using the inverse PCR protocol. pTM748 (*Aa*RseP-His_8_), pTM749 (active-site mutant E18Q), pTM750 (181-PA14-184) and pTM751 (235-PA14-236) were constructed by introducing a 1.5 kb fragment of the EcoRI/BamHI-digested pUC118-based plasmid (see above) into the same cloning site on pTWV228. All of the plasmids used in this study are listed in Supplementary Table S1.

### 2.2. Purification of the PA14-inserted PDZ tandem mutants in complex with the NZ-1 Fab   

Using the plasmids constructed above, the PA14-inserted PDZ tandem fragments were produced as N-terminal glutathione *S*-transferase (GST)-fusion proteins, in which a TEV protease recognition site was incorporated between the GST and PDZ tandem sequences, as reported previously (Hizukuri *et al.*, 2014 ▸). Each PA-inserted construct was overproduced in *E. coli* BL21(DE3) cells and purified from the cell lysate using Glutathione Sepharose 4B resin (Cytiva). The mutant fragment was cleaved from the GST portion through on-column digestion with TEV protease, and the released fragment, which contained two additional residues (Gly-Ser) upstream of the PDZ tandem, was further purified using cation-exchange chromatography (HiTrap SP HP, Cytiva) and size-exclusion chromatography (Superdex 200 Increase 10/300 GL, Cytiva). In parallel, the NZ-1 Fab was prepared by cleaving the NZ-1 antibody using papain and purifying as reported previously (Fujii *et al.*, 2016 ▸). NZ-1 was obtained from the Antibody Bank (http://www.med-tohoku-antibody.com/topics/antibody.htm) at Tohoku University, Miyagi, Japan. The purified PDZ tandem mutant was mixed with the NZ-1 Fab in a 2:1 molar ratio and was applied to size-exclusion chromatography to fractionate the complex. The final protein sample was concentrated by ultrafiltration.

### 2.3. Crystallization and data collection   

The initial crystallization conditions were screened using the Index screening kit from Hampton Research containing 96 conditions. 0.2 µl each of protein solution and reagent were dispensed into 96-well plates using a Gryphon robotic crystallization system (Art Robbins Instruments) and equilibrated against 60 µl reservoir solution by the sitting-drop vapor-diffusion method. Diffraction-quality crystals of the PDZ tandem (181-PA14-184) complexed with the NZ-1 Fab were generated from a crystallization buffer consisting of 20%(*w*/*v*) polyethylene glycol (PEG) 3350, 0.2 *M* potassium sodium tartrate. Diffraction-quality crystals of the PDZ tandem (235-PA14-236) complexed with the NZ-1 Fab were obtained from a crystallization buffer consisting of 10%(*w*/*v*) PEG 3350, 0.2 *M* l-proline, 0.1 *M* HEPES–Na pH 7.5. For each crystallization condition, cryoprotectant was prepared by mixing the crystallization buffer and ethylene glycol in a 4:1 volume ratio. All of the crystals were quickly soaked in the cryoprotectant and cooled in liquid nitrogen. X-ray diffraction data were collected using a PILATUS3 S 6M photon-counting pixel-array detector (Dectris) on BL-5A and BL-17A at Photon Factory (PF), Tsukuba, Japan. The data were processed and scaled with *XDS* (Kabsch, 2010 ▸) and *AIMLESS* (Evans & Murshudov, 2013 ▸). Diffraction intensities were converted to structure factors using programs from *CCP*4 (Winn *et al.*, 2011 ▸), where 5% of the unique reflections were randomly selected as a test set for calculation of the free *R* factor. Data-collection statistics are summarized in Table 1 ▸.

### 2.4. Crystallographic analysis   

For both of the co-crystals, initial phases were determined by the molecular-replacement method using *MOLREP* (Vagin & Teplyakov, 2010 ▸) in *CCP*4. Firstly, the Fv and constant regions of the NZ-1 Fab were assigned separately using the atomic coordinates of the NZ-1 Fab bound to the PA14 peptide (Fujii *et al.*, 2016 ▸; PDB entry 4yo0). Next, the PDZ-N and PDZ-C domains were searched for separately using the atomic coordinates of the *A. aeolicus* PDZ tandem (Hizukuri *et al.*, 2014 ▸; PDB entry 3wkl) with the NZ-1 Fab models fixed in the asymmetric unit. The assigned models were manually fitted into the electron-density map using *Coot* (Emsley *et al.*, 2010 ▸). The updated models were refined with *phenix.refine* (Afonine *et al.*, 2012 ▸) iteratively while monitoring the stereochemistry with *MolProbity* (Chen *et al.*, 2010 ▸). As reported in the previous study (Tamura *et al.*, 2019 ▸), the electron densities indicated that Asn150 side chain reacted with the main-chain amide group of Gly151 and formed a succinimide group in the PDZ tandem (181-PA14-184) mutant. Refinement statistics are summarized in Table 2 ▸. The atomic coordinates of the Fab complexes of the PDZ tandem (181-PA14-184) and (235-PA14-236) mutants have been deposited in the Protein Data Bank with accession codes 7cqc and 7cqd, respectively. Structural superpositions and r.m.s.d. calculations were performed by the pairwise alignment protocol using *LSQKAB* (Kabsch, 1976 ▸). Figures showing protein structures were prepared with *PyMOL* (version 2.3; Schrödinger).

### 2.5. *In vivo* cleavage assay of *Aa*RseP and its mutants   

The *in vivo* proteolytic activity of *Aa*RseP was analyzed using *E. coli* KK211 (Δ*rseA*, Δ*rseP*) cells (Kanehara *et al.*, 2002 ▸) as described previously (Akiyama *et al.*, 2015 ▸; Hizukuri *et al.*, 2017 ▸). *E. coli* KK211 (Δ*rseA*, Δ*rseP*) cells harboring pYH124 [HA-MBP-RseA(LY1)148] were transformed with the pKK11 plasmid (*Ec*RseP-His_6_-Myc; Kanehara *et al.*, 2001 ▸), pTM748 (*Aa*RseP-His_8_), pTWV228 or plasmids encoding their derivatives. M9 medium (without CaCl_2_; Miller, 1972 ▸) supplemented with 20 µg ml^−1^ of each of the 20 amino acids, 2 µg ml^−1^ thiamine, 0.4% glucose, 1 m*M* isopropyl β-d-1-thiogalactopyranoside (IPTG) and 5 m*M* cAMP was inoculated with transformed *E. coli* KK211 cells and grown at 30°C for 3 h. Proteins were precipitated by trichloroacetic acid (TCA) treatment and separated by Laemmli SDS–PAGE. Immunoblots with anti-HA, anti-His or anti-SecB antibodies were visualized using a Lumino LAS 4000 mini image analyzer (Cytiva) with ECL Prime Western Blotting Detection Reagents (Cytiva). Rabbit polyclonal anti-HA [HA-probe (Y-11), Santa Cruz Biotechnology] and anti-SecB (Miyake *et al.*, 2020 ▸) antibodies were used for immunoblotting. For the detection of His-tagged proteins, anti-His antibodies from the Penta-His HRP Conjugate Kit (Qiagen) were used.

### 2.6. Purification of the *Aa*RseP mutants and complex formation with the NZ-1 Fab   

For the EM analysis, the PA14-inserted *Aa*RseP mutant was overproduced in *E. coli* KK374 (Δ*rseA*, Δ*rseP*, Δ*degS*) cells (Akiyama *et al.*, 2004 ▸). *E. coli* KK374 cells transformed with the expression plasmids (Supplementary Table 1) were grown at 30°C to an OD_600_ of 0.7 in a medium containing 10 g Bacto tryptone, 5 g yeast extract and 10 g NaCl per litre supplemented with 50 µg ml^−1^ ampicillin, followed by the induction of overexpression with 0.1 m*M* IPTG and incubation at 30°C for an additional 4 h. The cells were harvested by centrifugation and lysed by sonication in 10 m*M* Tris–HCl pH 7.4, 150 m*M* NaCl. The cell lysates were centrifuged at 40 000*g* for 45 min at 277 K. Subsequently, the supernatant was further separated by ultracentrifugation at 200 000*g* for 90 min at 277 K. The membrane fraction collected as a precipitate was suspended in 10 m*M* Tris–HCl pH 7.4, 150 m*M* NaCl and was ultracentrifuged again under the same conditions. Finally, the precipitated membrane fraction was suspended in 10 m*M* Tris–HCl, 150 m*M* NaCl and the total protein was quantified using the bicinchoninic acid (BCA) assay. The suspension of the membrane fraction was diluted with the same buffer to adjust the protein concentration to 10 mg ml^−1^ using bovine serum albumin as a standard.

Membrane proteins were solubilized by adding the same volume of a solubilization buffer consisting of 40 m*M* Tris–HCl pH 8.0, 150 m*M* NaCl, 2% *n*-dodecyl-*N*,*N*-dimethyl­amine-*N*-oxide (DDAO) to the above-prepared suspension of the membrane fraction. After incubation at 277 K for 1 h, the mixture was ultracentrifuged at 210 000*g* for 90 min at 277 K. The supernatant was applied onto Ni–NTA agarose resin and the unbound fraction was washed out with a buffer consisting of 20 m*M* Tris–HCl pH 8.0, 300 m*M* NaCl, 20 m*M* imidazole, 0.05% DDAO. The resin was further washed with a buffer consisting of 20 m*M* Tris–HCl pH 8.0, 300 m*M* NaCl, 50 m*M* imidazole, 0.1% glyco-diosgenin (GDN) for detergent exchange. The *Aa*RseP mutant was eluted from the resin with a buffer consisting of 20 m*M* Tris–HCl pH 8.0, 300 m*M* NaCl, 250 m*M* imidazole, 0.1% GDN. The eluted mutant was then applied onto a Superdex 200 10/300 GL size-exclusion chromatography column (Cytiva) to isolate the monodisperse fraction of the *Aa*RseP mutant. Finally, the purified *Aa*RseP mutant was mixed with the NZ-1 Fab and was again subjected to size-exclusion chromatography to separate the complex fraction.

### 2.7. Negative-stain electron microscopy   

All purified samples were diluted to 1.0 µg ml^−1^. For negative-stain EM, 5 µl protein solution was applied onto glow-discharged, 600 mesh, carbon-coated grids. The grids were negatively stained with 2% ammonium molybdate, blotted with filter paper and air-dried. A JEM2200FS (JEOL) was operated at 200 kV to acquire EM images using a K2 camera (Gatan) in counting mode. Images were recorded at a magnification of 20 000× (1.98 Å per pixel) with a defocus of −0.5 to −2.0 µm. A movie of 50 frames was taken for each image, and motion correction was performed using *MotionCor*2 (Zheng *et al.*, 2017 ▸). The contrast transfer function was estimated by *Gctf* (Zhang, 2016 ▸). All subsequent processing was carried out using *RELION*3 (Zivanov *et al.*, 2018 ▸). Particles were selected using the *Relion Autopicker* and three rounds of 2D classification were performed to select particles for 3D reconstruction. The number of particles used for each reconstruction of the Fab-complexed *Aa*RseP (181-PA14-184) and (235-PA14-236) mutants are indicated in Fig. 7 and Supplementary Fig. S6, respectively. Fitting of the atomic coordinates of the Fab–PDZ complex into the 3D reconstruction model and figure preparation were performed with *UCSF Chimera* (Pettersen *et al.*, 2004 ▸).

### 2.8. Molecular-dynamics (MD) simulation   

Initial models were prepared by performing energy minimization using the crystal structures of the PA14-mediated complexes between the NZ-1 Fab and PDZ tandems from this study. For both of the two PDZ tandems, only the PDZ domain that contains the PA14 insertion was included in the initial model. For PDZ-N (181-PA14-184), residues 113–206, in which Gly113 and Ser114 were derived from the expression tag, were used in the simulation, while residues 207–292 were included for the PDZ-C (235-PA14-236) model. The dis­ordered loop regions in the NZ-1 Fab were built by *MODELLER* (Šali & Blundell, 1993 ▸). The succinimide in PDZ-N and the pyroglutamate at the N-terminus of the NZ-1 light chain were remodeled as aspartate and glycine and as glutamate, respectively. For the PA12-mediated complex, the crystal structure of PDZ-N (181-PA12-184) complexed with the NZ-1 Fab (PDB entry 6al1; Tamura *et al.*, 2019 ▸) was modified to prepare the initial model for the simulation according to the same procedure as described above. The model of the PDZ-N domain covered residues 113–206, while structures for the disordered loop and terminal regions were generated as described above. The solvent system around each Fab–PDZ complex was prepared by *Solution Builder* as implemented in *CHARMM-GUI* (Jo *et al.*, 2008 ▸; Lee *et al.*, 2016 ▸). The protonation state of histidine was calculated by *PROPKA* (Olsson *et al.*, 2011 ▸; Søndergaard *et al.*, 2011 ▸) as implemented in *PDB*2*PQR* (Dolinsky *et al.*, 2004 ▸) at pH 7. Missing H atoms were inserted using *Solution Builder*, and the N- and C-termini were set to NH_3_^+^ and COO^−^, respectively. The MD unit cell was set to a rectangular cell with a minimum edge distance of 10 Å between the protein and the walls of the cell. The cell was filled with the TIP3P water model (Jorgensen *et al.*, 1983 ▸) with 150 m*M* NaCl and added Na^+^ counter-ions.

The all-atom MD simulations were carried out using the *GROMACS* version 2016.3 MD program package (Abraham *et al.*, 2015 ▸; Pronk *et al.*, 2013 ▸) with the CHARMM36m force field (Huang *et al.*, 2017 ▸; MacKerell *et al.*, 1998 ▸, 2004 ▸) under periodic boundary conditions. The electrostatic interactions were handled by the smooth particle mesh Ewald method (Essmann *et al.*, 1995 ▸), and the van der Waals interactions were truncated by a switching function with a range of 10–12 Å. Bond lengths involving H atoms were constrained by the *P-LINKS* algorithm (Hess, 2008 ▸). According to the default setup of *CHARMM-GUI*, an energy minimization and a 125 ps equilibration run as an NVT ensemble with a 1 fs time step were executed before the production run. The production run was performed as an NPT ensemble with a 2 fs time step. The Nosé–Hoover scheme was used as the thermostat (Hoover, 1985 ▸, Nosé, 1984 ▸) and the Parrinello–Rahman approach was used as the barostat (Nosé & Klein, 1983 ▸; Parrinello & Rahman, 1981 ▸). The temperature and pressure were set to 300 K and 1 atm, respectively. The simulation length of the production run was 1 µs.

R.m.s.d. and r.m.s.f. values were calculated using snapshots extracted from each 1 µs simulation every 1 ns (1000 snapshots for each trajectory). For PDZ-N, the calculations were performed over the C^α^ atoms of residues 123–206 because residues 113–122 in the N-terminal region are not included in the PDZ fold and exhibited large fluctuations during the simulation compared with the remaining region of the PDZ-N domain. To estimate the structural change in the PDZ domains, r.m.s.d.s were calculated relative to the energy-minimized initial models of the respective PDZ domains, which were almost identical to the atomic models in the corresponding crystal structures, as shown in Fig. 5(*a*). To estimate the degree of fluctuation in the orientation of the PDZ domains relative to the NZ-1 Fab, r.m.s.f.s were calculated over the averaged structures of the respective complexes after aligning the snapshot models based on the V_H_ region (residues 20–130) in Figs. 5(*b*) and 5(*c*). In addition, r.m.s.d.s were calculated for the PDZ domains by aligning the snapshot models within a trajectory of a Fab–PDZ complex with the corresponding initial model based on the V_H_ region in Figs. 5(*e*) and 5(*f*) and Supplementary Fig. S4. Furthermore, principal component analysis (PCA) was carried out using the snapshot models aligned at the V_H_ region to separate out the characteristic movements in the fluctuations between the NZ-1 Fab and the respective PDZ domains. For the NZ-1 Fab–PDZ-C (235-PA14-236) complex, the two trajectories for complexes 1 and 2 were merged in the PCA calculation. The structural distribution was represented as a 2D normalized histogram of −ln(*Z*) plotted on a principal component map with a pixel size of 10 × 10 Å, where *Z* is the probability of a given conformation, as shown in Supplementary Figs. S7(*a*) and S7(*b*). Hierarchical clustering was implemented using the *MMTSB* tool (Feig *et al.*, 2004 ▸) to select a representative model with the maximum probability for each trajectory: the 605 ns snapshot for NZ-1 Fab–PDZ-N (181-PA14-184), the 317 ns snapshot for complex 1 of NZ-1 Fab–PDZ-C (235-PA14-236) and the 444 ns snapshot for complex 2 (Supplementary Figs. S7*a* and S7*b*).

## 3. Results   

### 3.1. Reduction of the structural change in the target by inserting the PA14 tag   

**Figure 2:**
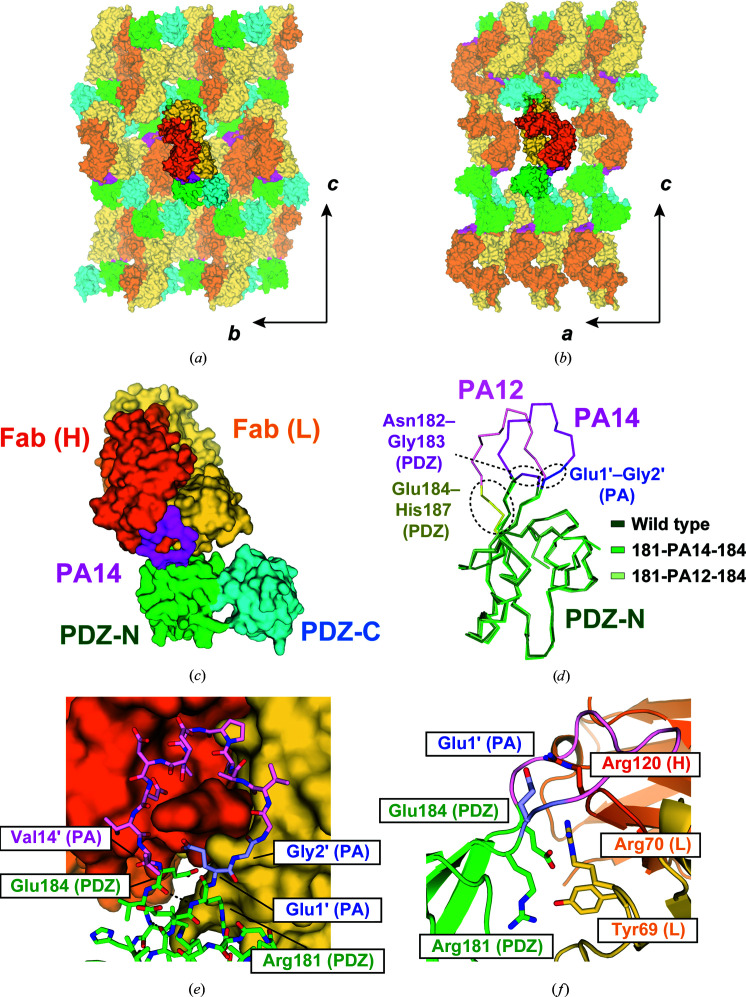
Crystal structure of PDZ tandem (181-PA14-184) complexed with the NZ-1 Fab. (*a*, *b*) Crystal packing in two different views. The crystallographic axes are indicated with arrows. The complex is shown as a surface model and the neighboring complexes in the crystal lattice are shown with partial transparency. The heavy chain and light chain of the Fab are colored dark and light orange, respectively. The PDZ-N and PDZ-C domains are colored green and cyan, respectively; the inserted PA14 tag is colored magenta. (*c*) Overall structure of the complex. The NZ-1 Fab docks to PDZ-N through PA14 and does not make direct contacts with PDZ-C within the complex. (*d*) Superposition of the C^α^ traces. The wild-type PDZ-N domain and the PA12- and PA14-inserted mutants are colored as in (*c*) and shown in light, medium and dark colors, respectively. The structures of the wild-type and PA12-inserted PDZ-N domains were extracted from previously determined crystal structures [PDB entries 3wkl (wild type) and 6al1 (PA12-inserted mutant)]. The turn residues replaced by the insertion (Asn182 and Gly183) and the inserted sequences (PA12 and PA14) are colored magenta, except for Glu1′ and Gly2′ in PA14, which are colored blue. In the PA12-inserted mutant, the junction residues highlighted in yellow (Glu184–His187) alter their conformations on PA12 insertion and complex formation. In contrast, the addition of Glu1′ and Gly2′ seemed to induce less structural change of the target PDZ-N domain, including the junction residues, in the PA14-inserted mutant. (*e*) Close-up view of the binding site. The NZ-1 Fab is shown as in (*c*). The PDZ-N domain and the inserted PA14 are shown as stick models and colored as in (*d*). Arg181 and Glu184 in the PDZ-N domain maintained the inter-strand hydrogen bond, as shown by dotted lines. (*f*) Residues at the binding interface. The binding interface from (*e*) is shown as a ribbon model and in profile. Residues where the side chains are presumed to form salt bridges or hydrogen bonds are displayed as stick models.

We first inserted the PA14 tag between βF and βA in the PDZ-N domain, where we previously inserted the PA12 tag, and optimized the linkers (Tamura *et al.*, 2019 ▸; Fig. 1 ▸). As βF–βA protrudes from the globular PDZ-N domain, it is highly possible that NZ-1 can bind to the inserted PA14 tag without steric hindrance. At the same time, this must be balanced with the need for a stiff linker to reduce conformational flexibility in the complex with the NZ-1 Fab. Hence, we deleted two protruding turn residues (Asn182 and Gly183) and inserted the PA14 tag between Arg181 and Glu184 [this mutant is referred to as PDZ tandem (181-PA14-184)]. Similar to the PA12-inserted mutants of the PDZ tandem in our previous study, purified PDZ tandem (181-PA14-184) was stable and monodisperse, indicating that the insertion of PA14 did not disrupt the folding of the PDZ tandem. The complex of PDZ tandem (181-PA14-184) with the NZ-1 Fab was also stable and produced crystals under several conditions. X-ray diffraction data were collected to 2.5 Å resolution (Table 1 ▸), and we assigned a PDZ tandem and an NZ-1 Fab in the asymmetric unit by molecular replacement. The crystal packing was maintained by contacts in both of the molecules (Figs. 2 ▸ *a* and 2 ▸ *b*). The electron densities were clear enough to build reliable models for both PDZ tandem (181-PA14-184) and the NZ-1 Fab (Fig. 2 ▸ *c*). However, the refined model of the PDZ-C domain showed higher temperature factors, probably because this domain made no direct contacts with the complex-forming NZ-1 Fab in the crystal (Table 2 ▸).

As we anticipated, the folding of PDZ-N was not disrupted by the PA14 insertion and complex formation with the NZ-1 Fab. All residues of the inserted PA14 tag were accommodated in the antigen-binding pocket of the NZ-1 Fab, where the 14 residues assumed a loop with closed ends, in other words a closed ring-like structure (Figs. 2 ▸ *d* and 2 ▸ *e*). For the 12 C-terminal residues of PA14 that constitute the PA12 tag, the structure was almost identical to that observed in the PA12-mediated complexes and in the PA14 peptide complexed with the NZ-1 Fab (Supplementary Figs. S1*b* and S1*c*). The 80 C^α^ atoms of PDZ-N, including Arg181 and Glu184, superposed onto those of the wild type with an r.m.s.d. of 0.750 Å. Arg181 and Glu184 at the insertion junctions formed inter-strand hydrogen bonds, as formed in wild-type PDZ-N (Fig. 2 ▸ *e*). The distance between the C^α^ atoms of Glu1′ and Val14′ (hereafter, residue numbers in the PA14 tag are indicated with a prime) at both ends of the PA14 tag was less than 7 Å, which was remarkably shorter than the separation caused by the PA12 insertion (Fig. 2 ▸ *d*). These observations suggest that labeling of NZ-1 with PA14 generally has less impact on the folding of the target than that with PA12.

Unexpectedly, the newly introduced Glu1′ residue in the inserted PA14 tag formed a salt bridge with Arg120 in the NZ-1 heavy chain (Fig. 2 ▸ *f*), whereas the same residue was highly mobile in the co-crystal structure of the NZ-1 Fab with the free PA14 peptide (Fujii *et al.*, 2016 ▸). Besides the inserted PA14 residues, only the junction residues in PDZ tandem (181-PA14-184) are involved in intermolecular interactions with the NZ-1 Fab (Fig. 2 ▸ *f*). Arg181 on PDZ-N at the N-terminal side formed a hydrogen bond with Tyr69 in the light chain, and Glu184 on PDZ-N is positioned to form a salt bridge with Arg70 in the light chain.

### 3.2. Insertion of the PA14 tag into a sterically hindered loop region   

**Figure 3:**
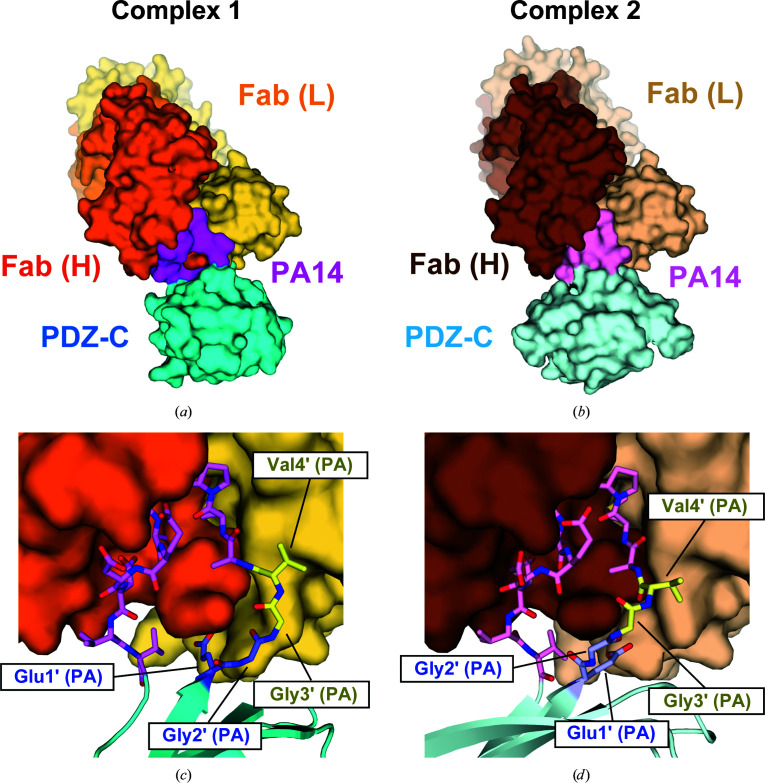
Crystal structure of PDZ tandem (235-PA14-236) complexed with the NZ-1 Fab. (*a*, *b*) The two complexes in the asymmetric unit, complexes 1 and 2, are shown as surface models. The heavy and light chains of NZ-1 Fab in complex 1 are colored dark and light orange, respectively, while those in complex 2 are colored dark and light brown, respectively. The inserted PA14 tags are colored dark and light magenta, respectively. Due to the disorder in the electron density, the PDZ-N domains were not included in the final model. (*c*, *d*) Close-up view of the binding site. The PDZ-C domain and the inserted PA14 residues are shown as ribbon and stick models, respectively. Glu1′-Gly2′ and Gly3′-Val4′ of PA14 are colored blue and yellow, respectively. The remaining ten C-terminal residues (Ala5′–Val14′) are colored magenta. The residues in complexes 1 and 2 are shown in dark and light colors, respectively.

We next tested whether the PA14 tag can be successfully inserted into multiple sites as a demonstration that PA14-mediated antibody labeling can serve as a generalizable strategy for structure determination. The insertion site for PDZ tandem (181-PA14-184) was chosen because this loop projects into solvent, but the context and orientation of the insertion site will be unknown in many practical applications of this method. To determine whether the PA14 tag could be inserted into a more sterically hindered site, we selected the βD–βE loop as the insertion site (Fig. 1 ▸). The βD and βE strands belong to a four-stranded β-sheet, and the turn residues (Asn235 and Gly236) are also included in the flat surface of the β-sheet. In contrast to NZ-1 labeling on a protruding loop, steric hindrance between NZ-1 and the target PDZ domain needs to be avoided in this case. Therefore, we inserted the PA14 tag between the turn residues without deletion, resulting in the mutant PDZ tandem (235-PA14-236). The complex of PDZ tandem (235-PA14-236) with the NZ-1 Fab was also successfully crystallized and diffraction data were collected to 3.2 Å resolution (Table 1 ▸). We assigned two complexes (referred to as complex 1 and 2) in the asymmetric unit by molecular replacement, but the electron densities for the PDZ-N domains were too weak to assign reliable models for this domain in either of the two complexes. Judging from the weak electron densities, the PDZ-N domains in both of the two complexes were exposed to the solvent and made little contribution to lattice formation in the crystal. Hence, the two structures of the NZ-1 Fab and the PA14-inserted PDZ-C domain were refined and included in the final model (Figs. 3 ▸ *a* and 3 ▸ *b* and Table 2 ▸).

**Figure 4:**
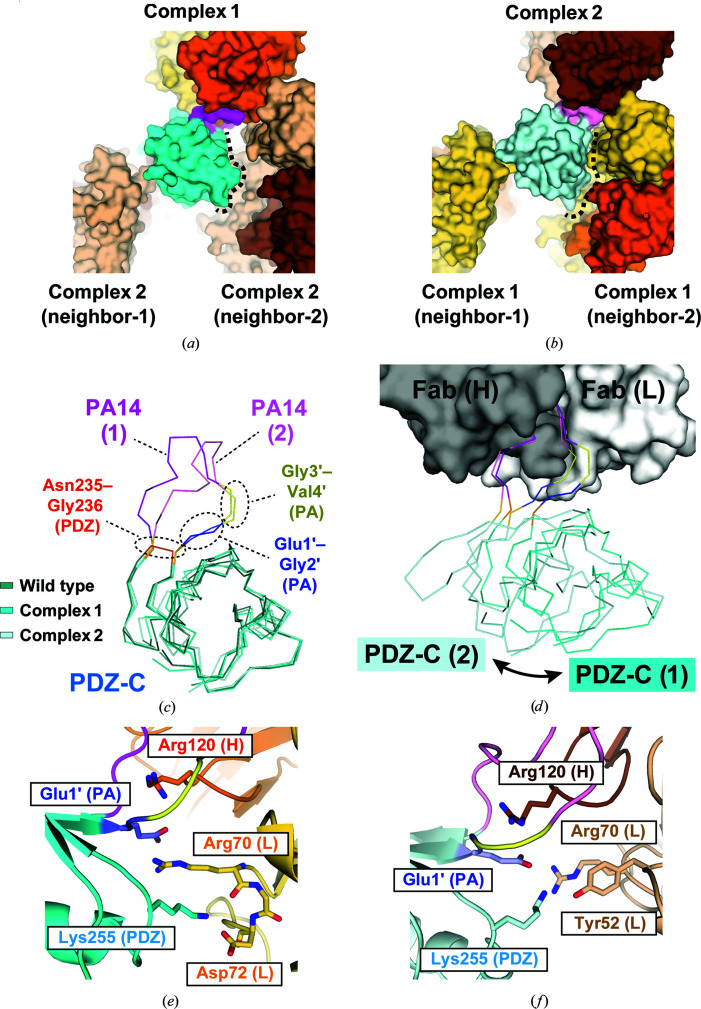
Comparison of the two conformations of PDZ tandem (235-PA14-236) complexed with the NZ-1 Fab. (*a*, *b*) Comparison of crystal contacts in complexes 1 and 2 with PA14-inserted PDZ-C domains. The structures are shown as in Figs. 3 ▸(*a*) and 3 ▸(*b*). In both complexes 1 and 2, the PDZ-C domains were in direct contact with two neighboring complexes, labeled neighbor-1 and neighbor-2. The PDZ-C domain of complex 1 had a relatively small contact area with neighbor-2 where solvent-accessible space was present between them. The relative arrangement of the NZ-1 Fab and PDZ-C in complex 1 did not appear to be affected by the crystal packing. In contrast, the PDZ-C domain of complex 2 intimately interacted with neighbor-2, indicating that the positioning of the PDZ-C domain with respect to the NZ-1 Fab was restricted by the crystal packing. (*c*) Superposition of the C^α^ traces. The wild-type PDZ-C domain (PDB entry 3wkl) and the PA14-inserted mutants in complexes 1 and 2 are shown in dark, medium and light colors, respectively. The PDZ-C domain is shown in cyan, while the insertion site (Asn235 and Gly236) is colored orange. The inserted PA14 tags are colored as in Figs. 3 ▸(*c*) and 3 ▸(*d*). (*d*) Superposition of the two complexes based on the NZ-1 Fab. The NZ-1 Fab in complex 1 is shown as a surface model. The heavy chain and light chains are colored gray and white, respectively. The interaction modes of Gly3′ and Val4′ of PA14 shown in yellow were different between the two complexes. As a result, the superposition showed a rigid-body rotational movement of the PDZ-C domain around the PA14-insertion site. (*e*, *f*) Residues at the binding interfaces of complexes 1 (*e*) and 2 (*f*). Residues presumed to form salt bridges or hydrogen bonds are displayed as stick models in the ribbon models.

As in PDZ tandem (181-PA14-184), the PA14 tag adopts a closed ring-like structure in both of the two complexes in the asymmetric unit. However, the conformations of the N-terminal residues were significantly different between them (Figs. 3 ▸ *c* and 3 ▸ *d* and Supplementary Figs. S1*d* and S1*e*). In all of the crystal structures that have been determined of the PA12 and PA14 tags so far, Gly3′ and Val4′ in the tag were located in proximity to Tyr52 and Tyr113 in the NZ-1 light chain. In complex 1, the conformations of Gly3′ and Val4′ were consistent with those in the known structures. In contrast, the two residues were largely separated from the NZ-1 light chain in complex 2, although they were still located inside the antigen-binding pocket. It appeared that the conformational change of these two residues was related to the crystal packing of the target PDZ-C domains (Figs. 4 ▸ *a* and 4 ▸ *b*). In complex 2, the PDZ-C domain closely contacted the neighboring Fab, resulting in a rigid-body reorientation of the PDZ-C domain relative to the complex-forming NZ-1 Fab. Despite the different interaction modes with the NZ-1 Fab, the main-chain structures of the PDZ-C domains in both complexes were consistent with that in the structure of the wild-type PDZ tandem without the PA14 insertion. The 81 C^α^ atoms of PDZ-C, including the junction residues Gly235 and Asn236, superposed onto to those of the wild type with r.m.s.d.s of 0.785 and 1.055 Å for complexes 1 and 2, respectively (Fig. 4 ▸ *c*). In contrast, a superposition of the Fv region between the two complexes showed a rotation of the PDZ-C domain by approximately 40° around the PA14-insertion site (Fig. 4 ▸ *d*), which was caused by the conformational change of Gly3′ and Val4′. It has previously been reported that the replacement of either Gly3′ or Val4′ by alanine had no significant effect on the affinity of the PA12 tag for NZ-1 (Fujii *et al.*, 2014 ▸). Presumably, these two residues tolerate conformational change to some extent as their side chains do not make a measurable energetic contribution to the binding affinity. Overall, these observations indicated that the PA14 tag could be inserted into this sterically hindered loop with less structural change in the target PDZ-C domain.

Concerning the specific interactions between the NZ-1 Fab and PDZ-C, Glu1′ formed salt bridges with Arg70 in the NZ-1 light chain as well as with Arg120 in the heavy chain in both complexes 1 and 2 (Figs. 4 ▸ *e* and 4 ▸ *f*). In addition to the PA14 residues, Lys255 in PDZ-C also formed intermolecular contacts with the NZ-1 Fab, although Lys255 made different contacts in the two complexes. In complex 1, the amino group of Lys255 formed hydrogen bonds to the carbonyl group of Arg70 in the light chain as well as the side-chain carboxyl group of Asp72 in the light chain. In complex 2, the same amino group formed a hydrogen bond with the hydroxyl group of Tyr52 in the light chain. Thus, the interaction mode of Lys255 is also affected by the reorientation of PDZ-C relative to the NZ-1 Fab.

### 3.3. Molecular-dynamics simulations to analyze conformational variation in PA14-mediated Fab–PDZ complexes   

**Figure 5:**
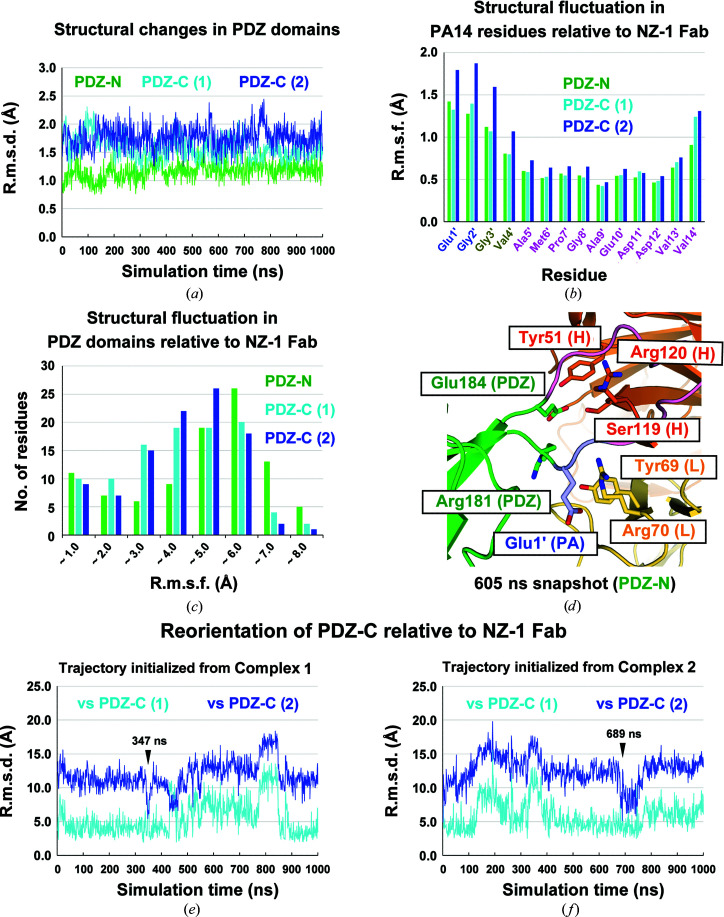
MD simulation of NZ-1 Fab–PDZ complexes. (*a*) Trajectories for the target PDZ structures. R.m.s.d.s were calculated for the snapshot models relative to the initial model to estimate the structural changes in the PDZ domains during the simulation trajectories. The inserted PA14 residues and the flexible N-­terminal region upstream of the PDZ-N domain were excluded from the calculation. R.m.s.d.s are plotted for trajectories of the PDZ-N domain (green) and for the PDZ-C domain in both complex 1 (cyan) and complex 2 (blue). (*b*) Structural fluctuations in the residues within the PA14 tag. In the r.m.s.f. calculations, the complex models within each trajectory were aligned with the initial model based on the V_H_ region of the NZ-1 Fab, and the structural fluctuation from the averaged structure was calculated for each residue in PA14. (*c*) Histogram of PDZ-domain residues binned by r.m.s.f. values calculated relative to the NZ-1 Fab. Similar to the process for calculating r.m.s.f. values in (*b*), the snapshot models within a trajectory were aligned with the averaged structure at the respective V_H_ regions for each of the three complexes. Subsequently, the fluctuation in the atomic coordinates was calculated for each residue. (*d*) The representative snapshot of PDZ-N (181-PA14-184) complexed with NZ-1 Fab from the trajectory. The snapshot at 605 ns, representing the most frequently observed conformation, is shown as a ribbon model. The PDZ-N domain is colored green. PA14 residues are colored light magenta, except for Glu1′ and Gly2′ at the N-terminus, which are colored light blue. The heavy and light chains in NZ-1 are colored dark and light orange, respectively. Glu1′ in PA14 was separated both from Arg120 in the NZ-1 heavy chain and from Arg70 in the NZ-1 light chain. Arg181 was also separated from Tyr69 in the light chain. Glu184 in PDZ-N interacted with Tyr51 and Ser119 in the NZ-1 heavy chain as opposed to the interaction with Arg70 in the NZ-1 light chain in the crystal structure (see Fig. 2 ▸ *f*). (*e*, *f*) Reorientation of the PDZ-C domains relative to the NZ-1 Fab in the MD simulations initialized from complexes 1 (*e*) and 2 (*f*). For both of the calculations, the snapshot models were aligned with the initial models of complexes 1 and 2 based on the V_H_ region, and the r.m.s.d.s for the PDZ-C domains were calculated relative to the initial models. The r.m.s.d.s calculated using complexes 1 and 2 as references are shown in cyan and blue, respectively. The conformations of the 347 ns snapshot in (*e*) and the 689 ns snapshot in (*f*) are similar to that of complex 2, as analyzed in Supplementary Fig. S7(*c*).

For the broadest application of this method, a fixed conformation of the Fab is desirable. Because crystallization may artificially fix the conformation of the complex subunits, we turned to molecular-dynamics (MD) simulations to characterize the complex stability and intermolecular motions in the NZ-1 Fab–PDZ complexes. For all complex structures, only the PDZ domain that contains the PA14 insertion was included in the complex model. For the 235-PA14-236 mutant, the structures of both complexes 1 and 2 were simulated in individual MD trajectories. During all simulations, the inserted PA14 tag maintained the closed ring-like conformation inside the antigen-binding pocket, and the PDZ domains never dissociated from the NZ-1 Fab (Supplementary Fig. S2). In all cases, the structures of the PA14-inserted PDZ domains were relatively rigid over 1 µs of simulation, and the r.m.s.d.s relative to the initial models were ∼2 Å, excluding the inserted PA14 residues (Fig. 5 ▸ *a*). The results of the simulations suggested that the impact of the PA14 insertion on the folding of the target PDZ domain was relatively small. Focusing on the inserted PA14 tag, the four N-terminal residues Glu1′–Val4′ showed relatively high root-mean-square fluctuations (r.m.s.f.s; Fig. 5 ▸ *b*), which coincides with the observation that the conformation of Gly3′ and Val4′ was dependent on the crystal packing in PDZ tandem (235-PA14-236) with the NZ-1 Fab (Figs. 3 ▸ *c* and 3 ▸ *d*). As a comparison, we also performed an MD simulation on our previously reported structure of the PA12-inserted PDZ-N mutant [PDZ-N (181-PA12-184)] complexed with the NZ-1 Fab (Tamura *et al.*, 2019 ▸). During the simulation, the inserted PA12 residues and junction residues showed high r.m.s.f.s, while the rest of the target PDZ-N domain was stable (Supplementary Fig. S3*a*). In particular, the four C-terminal residues of PA12, Asp11′–Val14′, fluctuated to a larger extent (Supplementary Figs. S3*b*–S3*d*). The conformation of the junction residues also seemed to be unstable in the PA12-inserted mutant, where the distance between Arg181 and Glu184 in the heavy chain fluctuated greatly during the simulation (Supplementary Fig. S3*e*). These observations suggested that in the context of a loop insertion, the PA14 tag binds to the NZ-1 Fab more stably than the PA12 tag.

With respect to the subunit arrangement in the complex, PDZ-N (181-PA14-184) with the NZ-1 Fab was more flexible even though there was only one conformation in the asymmetric unit of the crystal structure. PDZ-N (181-PA14-184) showed higher r.m.s.d. and r.m.s.f. values than PDZ-C (235-PA14-236) in the simulations where the structures of the complex were aligned based on the variable region of the heavy chain (V_H_ region) (Figs. 5 ▸ *c*, 5 ▸ *e*, 5 ▸ *f* and Supplementary Fig. S4). At 358 ns in the 1 µs simulation, PDZ-N (181-PA14-184) showed the highest r.m.s.d. value (∼19 Å) relative to the initial model (Supplementary Figs. S2*a* and S4). In fact, almost no hydrogen-bonding pairs were observed between the NZ-1 Fab and the PDZ-N domain during the simulation, except for those formed by the inserted PA14 tag. Additionally, Glu1′ in PA14 became separated from the NZ-1 Fab, whereas this residue was placed close to Arg120 in the heavy chain in the crystal structure (Fig. 5 ▸ *d*). For PDZ-C (235-PA14-236) complexed with the NZ-1 Fab, the preferential conformations were more similar to complex 1 than to complex 2 (Figs. 5 ▸ *e* and 5 ▸ *f*). In the simulation initialized from complex 2, the model immediately sampled conformations that were more similar to complex 1, although conformations similar to complex 2 also appeared as a minor population (Fig. 5 ▸ *f*). Comparing the snapshot structures, we found that Lys255 in PDZ-C formed hydrogen bonds to residues in the NZ-1 light chain such as Asp72 in conformations similar to complex 1 (Supplementary Fig. S5*a*). In contrast, Lys255 was separated from Asp72 in conformations similar to complex 2 (Supplementary Fig. S5*b*). Furthermore, the contribution of Glu1′ in PA14 to complex formation depended on the PA-insertion sites. Glu1′ in PDZ-C (235-PA14-236) formed a hydrogen bond to Arg70 in the NZ-1 heavy chain in 72.6% of the snapshots on the trajectory, whereas Glu1′ in PDZ-N (181-PA14-184) reoriented into the solvent and away from the interaction with Arg120 in the NZ-1 heavy chain in the MD simulation, as mentioned above.

### 3.4. Preparation of PA14-inserted full-length *Aa*RseP for NZ-1 labeling   

Using our improved NZ-1 labeling technique, we next attempted to determine the location and orientation of the two PDZ domains in the context of the full-length *Aa*RseP using antibody-assisted negative-stain EM. Although no high-resolution 3D structural data have been generated to date for any full-length RseP orthologue, we have previously estimated the spatial arrangement of the PDZ domains using biochemical methods (Hizukuri *et al.*, 2014 ▸). Specifically, we performed chemical modification analysis to estimate the solvent accessibility of the surface residues of *Ec*RseP based on structural data for the PDZ tandem fragment from X-ray crystallo­graphy and small-angle scattering analysis. These previous results suggested that the two PDZ domains adopted a clam-like structure and that the putative ligand-binding grooves in the PDZ domains would be inaccessible in the context of the full-length protein on the cell membrane. Based on this estimation, the two PA14-insertion sites tested in the present study were presumed to project into solvent because they were located on the back side of the putative ligand-binding grooves. Therefore, it was expected that PA14 insertion would not destabilize the structure of the full-length protein in both mutants.

**Figure 6:**
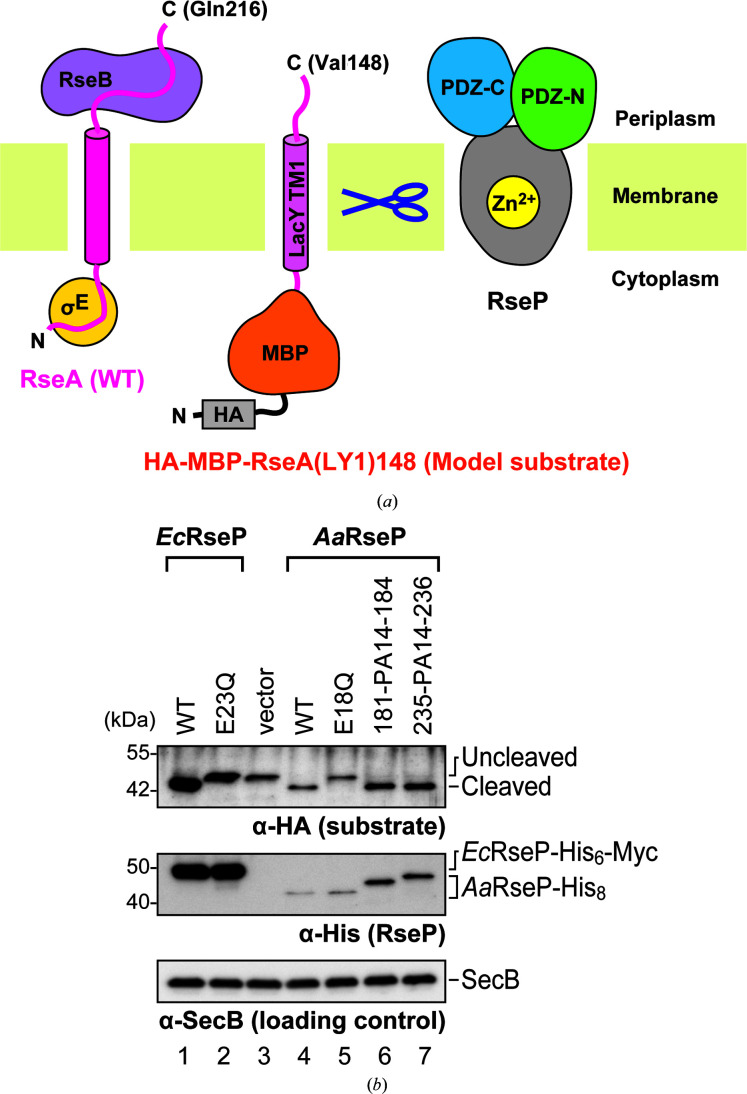
*In vivo* cleavage of a model substrate by wild-type *Aa*RseP and PA-inserted mutants. (*a*) Design of the model substrate. The wild-type RseA from *E. coli* is a type II membrane protein composed of 216 amino-acid residues. The full-length RseA binds σ^E^ and RseB in the cytoplasmic and periplasmic domains, respectively. DegS cleaves RseA at the C-terminal side of Val148 and liberates RseB. The model substrate, HA-MBP-RseA(LY1)148, contains the first transmembrane region of lactose permease (LacY TM1; LY1) with a recombinant cytoplasmic domain composed of HA-tagged MBP. The C-terminal periplasmic domain was truncated at Val148 to mimic the DegS-cleaved product. (*b*) Immunoblotting with anti-HA antibody detected the model substrate. The full-length model substrate appeared at the ‘Uncleaved’ position. ‘Cleaved’ indicates the model substrate that was cleaved by RseP within the membrane, as illustrated in (*a*). Immunoblotting with anti-His antibody detected His-tagged *Ec*RseP, *Aa*RseP and their derivatives. Immunoblotting of the cytoplasmic protein SecB serves as a loading control. Molecular-size marker positions are shown in kDa on the left.

We therefore performed negative-stain EM structural analysis on full-length *Aa*RseP constructs with PA14 tag insertions. For these full-length constructs, we used the same insertion sites as used for the PDZ tandem fragments and produced *Aa*RseP (181-PA14-184) and *Aa*RseP (235-PA14-236) for PDZ-N and PDZ-C, respectively. After confirming that both mutants accumulated in the *E. coli* membrane, we attempted to examine whether or not the PA14 insertion affected proteolytic activity in *Aa*RseP. However, no physiological substrates have yet been identified for *Aa*RseP. It is known that *Ec*RseP cleaves the anti-sigma factor RseA (Alba *et al.*, 2002 ▸; Kanehara *et al.*, 2002 ▸), but no orthologue of RseA has been identified in the *A. aeolicus* genome. Looking for an alternative, we discovered that *Aa*RseP is able to cleave a model substrate for *Ec*RseP derived from RseA (Fig. 6 ▸ *a*). In this model substrate, the periplasmic domain of RseA is fused to the first transmembrane region of lactose permease (LY1) with a recombinant cytoplasmic domain containing hemagglutinin (HA)-tagged maltose-binding protein (MBP). Furthermore, the C-terminal periplasmic region of the model substrate was truncated at Val148 because *Ec*RseP performs the intramembrane proteolysis only after the periplasmic region of RseA has first been cleaved by DegS (Alba *et al.*, 2002 ▸; Kanehara *et al.*, 2002 ▸). Hence, the model substrate mimics the DegS-cleaved product and can be detected by anti-HA antibody labeling. This model substrate is referred to as HA-MBP-RseA(LY1)148 (Hizukuri & Akiyama, 2012 ▸). When co-expressed with either wild-type *Aa*RseP or *Ec*RseP, the band for HA-MBP-RseA(LY1)148 shifts compared with the vector control containing neither *Aa*RseP nor *Ec*RseP. We also observed that the band for HA-MBP-RseA(LY1)148 does not shift when the catalytically important glutamate residue of the HE*XX*H sequence that is conserved among the site-2 protease family of intramembrane proteases was mutated to a glutamine in either *Ec*RseP or *Aa*RseP, which is consistent with intramembrane proteolysis of HA-MBP-RseA(LY1)148 by the respective RsePs. These observations confirm for the first time that *Aa*RseP is functionally orthologous to *Ec*RseP and belongs to the site-2 protease family. Finally, both *Aa*RseP (181-PA14-184) and *Aa*RseP (235-PA14-236) were also able to cleave HA-MBP-RseA(LY1)148 at comparable levels to wild-type *Aa*RseP (Fig. 6 ▸ *b*), indicating that the PA14 insertion did not abrogate proteolytic activity.

### 3.5. Antibody-assisted negative-stain EM of full-length *Aa*RseP   

**Figure 7:**
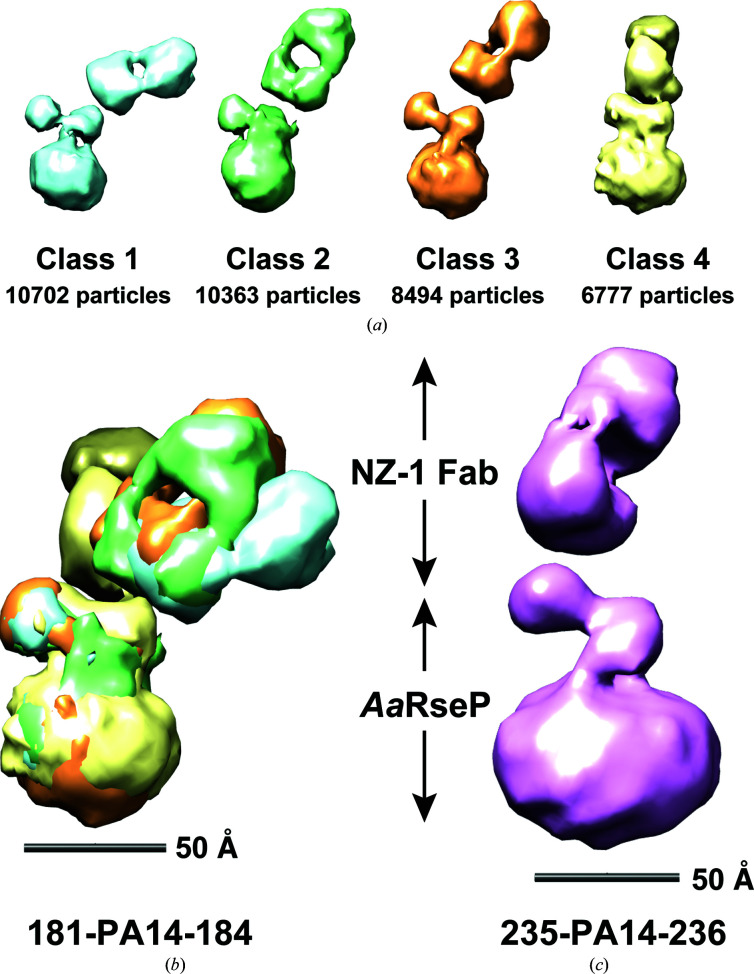
Antibody-assisted negative-staining EM analysis of PA14-inserted full-length *Aa*RseP. (*a*) 3D reconstruction models of *Aa*RseP (181-PA14-184) complexed with the NZ-1 Fab. Four different 3D reconstructions from the 2D class average images are shown in different colors. The numbers of particles used for the reconstruction are indicated below each model. (*b*) Superposition of the 3D reconstructions. The four models in (*a*) were aligned based on the putative *Aa*RseP region. The NZ-­1 Fabs appear to contact *Aa*RseP at almost the same point in each model. (*c*) 3D reconstruction model of *Aa*RseP (235-PA14-236) complexed with the NZ-1 Fab. The most well averaged class is drawn as a representative.

We next overproduced the PA14-inserted *Aa*RseP mutants in *E. coli* and purified them using immobilized metal-affinity and size-exclusion chromatography. We fractionated monodisperse mutants solubilized with glyco-diosgenin (GDN) detergent and mixed them with the NZ-1 Fab. We then subjected the mixture to size-exclusion chromatography again to fractionate the stable complex. Subsequently, the purified complex was negatively stained with ammonium molybdate for EM single-particle analysis. For *Aa*RseP (181-PA14-184) complexed with the NZ-1 Fab, four different classes of 2D averages were reconstructed into 3D models (Fig. 7 ▸ *a*). In each model, the putative Fab fragment was identified by a characteristic ellipsoidal shape with a hole at the center. The remaining part is constituted of a sphere and two protrusions, and presumably corresponds to the detergent-solubilized TM domain and the two PDZ domains of *Aa*RseP. Structural alignment of the four 3D models with the putative *Aa*RseP molecule showed that the relative orientation of the NZ-1 Fab among the models hinges around a fixed point (Fig. 7 ▸ *b*). This hinge-like variation indicates that the contacting points, namely the locations of the PA14 insertion sites, are consistent among the four classes despite the variable orientation of the NZ-1 Fab. It was therefore highly possible that the arrangement of the PDZ-N domain was also consistent among the four classes. In fact, the abovementioned MD simulation also demonstrated that the orientation of the NZ-1 Fab relative to PDZ-N (181-PA14-184) fluctuated, while the folding of the target PDZ-N domain was maintained throughout the simulation time. For *Aa*RseP (235-PA14-236) complexed with the NZ-1 Fab, the 3D reconstruction also produced four different classes. In each of these four classes, however, the densities presumed to be the NZ-1 Fab were located in a common location relative to the spherical profile, although the resolutions of the EM maps were relatively low for three classes (Supplementary Fig. S6). This observation is also consistent with the result from the MD simulation, where the orientation of the NZ-1 Fab relative to PDZ-C (235-PA14-236) was more fixed compared with PDZ-N (181-PA14-184). In the most well averaged class the distinctive shape of the NZ-1 Fab was recognized, while two protrusions were again identified on the spherical part (Fig. 7 ▸ *c*). Taken together, we could successfully obtain 3D structural data for the full-length RseP orthologue for the first time, which could be utilized to analyze the spatial arrangement of the two PDZ domains.

### 3.6. Approximation of the domain arrangement in full-length *Aa*RseP   

**Figure 8:**
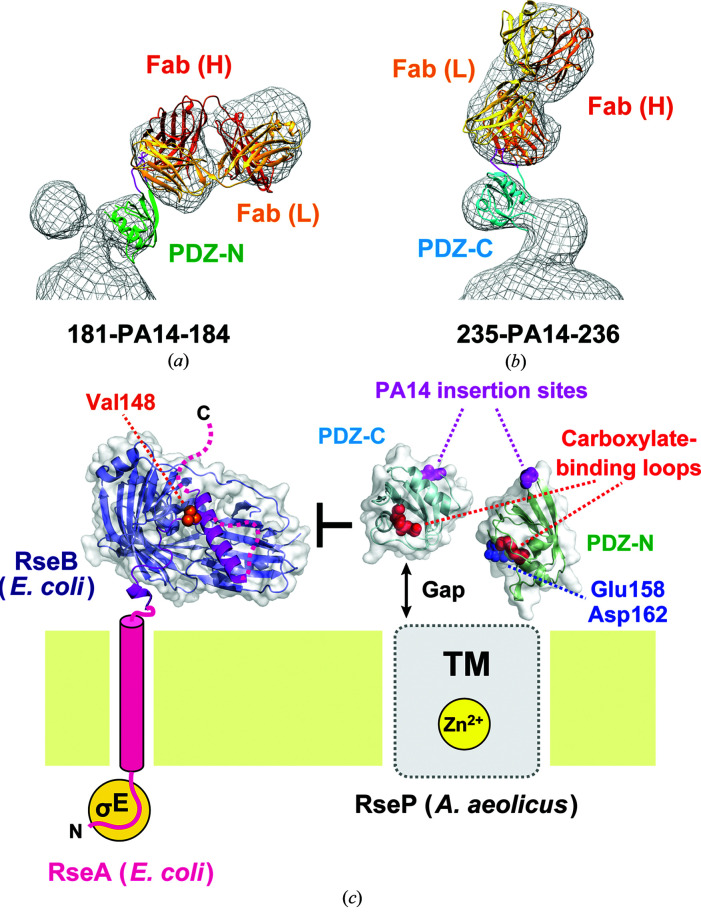
Estimation of the arrangement of the two PDZ domains in full-length *Aa*RseP. (*a*, *b*) Structural alignment of the Fab–PDZ models onto the 3D reconstruction. (*a*) The 605 ns snapshot in the MD trajectory of the NZ-1 Fab–PDZ-N (181-PA14-184) pair, whose conformation was most frequently sampled during the MD simulation, was fitted into the 3D reconstruction model of class 1 shown in Fig. 7 ▸(*a*) based on the position of the Fv region. (*b*) The 347 ns snapshot, which showed a similar conformation to that of the complex 2 structure, in the MD trajectory calculated from the complex 1 structure of the NZ-1 Fab–PDZ-C (235-PA14-236) pair was fitted into the 3D reconstruction model shown in Fig. 7 ▸(*c*) based on the position of the Fv region. (*c*) Structural model of size exclusion. The approximate arrangement of the two PDZ domains from *Aa*RseP is shown together with the model of RseA from *E. coli* to help understand the size-exclusion mechanism. The crystal structure of the periplasmic domain of RseA in complex with RseB (PDB entry 3m4w) is drawn in the RseA model. RseB is shown as a blue ribbon model with a partial transparent surface. The RseA periplasmic domain is shown as magenta ribbon model, and the side chain of Val148 is highlighted with an orange sphere model. The atomic model of the PDZ tandem from *Aa*RseP was built based on the structural alignments in (*a*) and (*b*). Firstly, the two 3D reconstruction models were aligned based on the *Aa*RseP molecule composed of a sphere and two protrusions. Next, the position of the PA14-inserted PDZ-N and PDZ-C domains were aligned onto the respective 3D models as described in (*a*) and (*b*). Finally, the crystal structures of wild-type PDZ-N and PDZ-C domains were independently aligned onto the models of the PA14-inserted mutants. The aligned models of PDZ-­N and PDZ-C were merged to build the entire PDZ tandem model. The PA14-insertion sites and the carboxylate-binding loops within the ligand-binding grooves are highlighted with magenta and red sphere models, respectively. Glu158 and Asp162, shown as blue sphere models, were modeled to be close to the transmembrane region based on the structural alignment. The corresponding residues in *Ec*RseP were estimated to be membrane-proximal residues based on protection from chemical modification. In both 3D reconstruction models the PDZ-C domain was separated from the TM domain by a gap, which might suppress the entry of the RseB-bound full-length RseA to the active center as the size-exclusion filter.

We then attempted to superpose the representative models of the Fab–PDZ complexes obtained from MD simulation onto the 3D reconstruction models of full-length *Aa*RseP with the NZ-1 Fab from EM analysis. To select the conformation that appeared with high probability as a representative conformation, we performed principal component analysis (PCA) for the snapshots within the MD trajectories. For the 181-PA14-184 mutant, the representative model obtained from PCA (605 ns snapshot in Fig. 5 ▸ *d* and Supplementary Fig. S7*a*) fitted class 1 of the 3D reconstruction model best. Aligning the representative model and the 3D reconstruction at the Fv region resulted in good overlap between the PDZ-N model and one of the protruding lobes of the EM map (Fig. 8 ▸ *a*).

For the 235-PA14-236 mutant, we first attempted to perform a structural alignment onto the 3D reconstruction using the representative models of the PDZ-C domain complexed with the NZ-1 Fab obtained from PCA (Supplementary Figs. S5*a* and S7*b*). However, neither the representative models calculated from complex 1 nor complex 2 fitted the 3D reconstruction well. Superposition of the NZ-1 Fab region positioned the βA–βB loop of the PDZ-C domain outside the EM map in both models (Supplementary Figs. S8*a* and S8*c*). These two models adopted conformations similar to that of complex 1 from the crystal structure. In fact, the atomic model of complex 1 also fitted the 3D reconstruction poorly (Supplementary Figs. S8*a* and S8*c*). The PDZ-C domain in complex 1 was located closer to the NZ-1 Fab than that in complex 2. In most trajectory snapshots, the PDZ-C domains were also located relatively close to the NZ-1 Fab, as observed in complex 1 (Supplementary Figs. S2*b* and S2*c*). In contrast, the PDZ-C domain in complex 2 was relatively separated from the NZ-1 Fab, and structural alignment of complex 2 onto the 3D reconstruction resulted in a better overlap of the PDZ-C domain with the protruding lobe in the EM map (Supplementary Figs. S8*b* and S8*d*). Within the trajectories initialized from both complexes 1 and 2, there were minor populations of snapshots with conformations similar to that in complex 2 (Supplementary Fig. S7*b*). In these snapshots, the PDZ-C domains were relatively separated from the NZ-1 Fab. In addition, comparison of the snapshot models indicated that Lys255 in PDZ-C and Asp72 in the NZ-1 light chain were not close enough to form a hydrogen bond in the conformations similar to that in complex 2. In fact, the r.m.s.d. of the snapshot model relative to complex 2 showed a negative correlation with the distance between Lys255 and Asp72 (Supplementary Fig. S7*c*). To analyze the structural features of the MD models that fit the 3D reconstruction, we selected one snapshot that had both a low r.m.s.d. relative to complex 2 and a large distance between Lys255 and Asp72 for each of the trajectories initialized from complexes 1 and 2 (Supplementary Figs. S5*b* and S7*c*). According to the PC surface, the selected snapshots were intermediate conformations between complexes 1 and 2 (Supplementary Fig. S7*b*). As a result, both of the two selected MD models (the 347 ns snapshot from complex 1 and the 689 ns snapshot from complex 2) seemed to fit the density similarly to complex 2 (Fig. 8 ▸ *b* and Supplementary Figs. S8*b* and S8*d*). From these observations, we concluded that the hydrogen bond between Lys255 in PDZ-C and Asp72 in the NZ-1 light chain is broken in the 3D reconstruction model of the complex.

Finally, combination of the two EM reconstructions enabled us to approximate the spatial arrangement of the PDZ tandem in the context of the full-length protein (Fig. 8 ▸ *c*). Firstly, the two EM maps were aligned based on the position of the spherical part and two protrusions. Next, structural alignments of the MD models onto the EM maps were performed for the Fab–PDZ-N complex (the 605 ns snapshot) and the Fab–PDZ-C complex (the 347 ns snapshot from complex 1), respectively. Subsequently, the crystal structure of the wild-type PDZ-N and PDZ-C domains were aligned onto the MD models and merged to build the entire PDZ tandem model. In the resultant model, the putative ligand-binding grooves of both of the PDZ domains pointed towards the TM domain containing the active center. In addition, both of the 3D reconstruction models commonly indicated that a gap was present between the PDZ-C domain and the TM domain, whereas it seemed that the PDZ-N domain directly contacts the spherical detergent micelle covering the TM domain.

## 4. Discussion   

Antibody labeling has been widely used in the structural analysis of protein molecules and complexes. The use of inserted exogenous epitopes has the potential to expand the applicability of antibody labeling, even to cases where no antibodies have been established for the target protein. In fact, antibody-labeling methods for membrane proteins mediated by fusion with a thermostabilized variant of apocyto­chrome *b*_562_ have been reported very recently (Miyagi *et al.*, 2020 ▸; Mukherjee *et al.*, 2020 ▸). Our method provides a new option for antibody labeling through exogenous epitopes. However, the structural integrity of the target protein must be maintained for the resulting structural model to be relevant in any of the methods. In our previous study of antibody-assisted structural analysis with NZ-1 labeling, the inserted PA12 tag caused a large structural change around the insertion site in the target protein. In the present study, we attempted to circumvent the deformation that PA12 caused by utilizing the PA14 tag that possesses a Glu–Gly pair of residues upstream of PA12. We anticipated that the two additional residues would act as a buffer region to reduce the structural change in the target. We observed that two different PA14-inserted PDZ tandem mutants both produced co-crystals with the NZ-1 Fab, which indicated that stable antibody–target complexes were successfully formed through the inserted PA14 tag. In these crystal structures, the loop-like conformations of the inserted PA14 tags were almost the same as those observed in the co-crystals of PA12-inserted PDZ tandems complexed with the NZ-1 Fab as well as the PA14-free peptide complexed with the NZ-1 Fab (Supplementary Fig. S1). Besides, the addition of Glu–Gly residues at the N-terminus resulted in a closed ring-like conformation for the entire 14-residue epitope when recognized by the NZ-1 Fab in both cases. In these conformations, the ends of the PA14 tag were located less than 7 Å apart based on the C^α^–C^α^ distance of Glu1′ and Val14′. Due to this structural property of the inserted PA14 tag, no significant structural changes were observed in the target PDZ domains in comparison to the wild-type structure without the PA14 insertion. In the previous study on inserting the PA12 tag, the insertion point needed to be optimized to produce a rigid complex with the NZ-1 Fab. In particular, eliminating residues that undergo structural change upon complex formation is quite important. However, prior structural data for the target are necessary for such an optimization. In contrast, we expect that the PA14 tag, adopting a closed ring-like structure, can be inserted into a target with fewer optimization steps. In addition, the MD simulations suggested that an inserted PA14 tag exhibits less structural fluctuation inside the NZ-1 antigen-binding pocket than an inserted PA12 tag. Taken together, we conclude that the PA14 tag is more suitable both for insertion into the target and for complex formation with NZ-1 compared with the previously used PA12 tag. This result opens the possibility that the PA14 tag can be inserted into other proteins with varying structural features.

In the present study, we also demonstrated that NZ-1 labeling via an inserted PA14 tag can be applied to negative-stain EM for structural analyses, as demonstrated using the PDZ tandem in *Aa*RseP. As the PDZ tandem is thought to play a pivotal role in substrate discrimination in RseP, structural data on the spatial arrangement of the PDZ domains should help to delineate the regulatory mechanism of intramembrane proteolysis. Our previous chemical modification analysis has suggested that the putative ligand-binding grooves of the two PDZ domains form a pocket-like space sitting just above the active center of RseP that is sequestrated within the membrane (Hizukuri *et al.*, 2014 ▸). The spatial arrangement approximated by EM analysis is fully consistent with the chemical modification analysis, wherein the putative ligand-binding grooves indeed point towards the TM region. In the chemical modification of *Ec*RseP, Asp162 and Leu167 in the αB helix were assigned as membrane-proximal residues. In the PDZ tandem model of *Aa*RseP, the corresponding residues, Glu158 and Asp162, were located at the interface with the TM domain (Fig. 8 ▸ *c*). As structural changes in the PDZ domains were not induced by the use of a PA14 insertion, we conclude that the arrangement of the PDZ tandem reflects the native state. The biochemical analysis confirmed that within the cell membrane the PA14-inserted mutants at least have a proteolytic activity similar to that of the wild type. In our previous study, we proposed that the pocket-forming PDZ tandem serves as a size-exclusion filter that only accommodates the RseA substrate for cleavage after pre-cleavage by DegS (Hizukuri *et al.*, 2014 ▸). Based on the 3D reconstruction model, the PDZ-C domain was separated from the TM domain. The degree of separation may control substrate discrimination (Fig. 8 ▸ *c*). The structural data from our present hybrid analysis will advance biochemical analyses in the investigation of the ‘size-exclusion model’.

The present study has raised a new issue to be solved when designing PA14-insertion sites for EM structural analysis assisted by NZ-1 labeling. While antibody labeling has applications in negative-stain EM (Boisset *et al.*, 1993 ▸, 1995 ▸), the impact of this method has been particularly pronounced in 3D structure determination by cryo-EM. Although cryo-EM techniques have been drastically improved through the ‘resolution revolution’ of recent years (Kühlbrandt, 2014 ▸), it is still difficult to determine high-resolution structures of small proteins due to the weak signal from individual particles in the EM images (Rubinstein, 2007 ▸). Antibody labeling is expected to resolve this size-limitation problem both by increasing the particle size and by facilitating accurate image alignment (Wu *et al.*, 2012 ▸), as has already been exemplified by recent cryo-EM structure determinations (Kim *et al.*, 2019 ▸; Coleman *et al.*, 2019 ▸). Similarly to as in X-ray crystallography, more rigid complexes with Fabs are however desirable in order to determine high-resolution structures by cryo-EM. While lattice contacts can stabilize the arrangement of proteins in a complex to some extent for X-ray crystallo­graphy, individual complex particles in EM images may display more structural heterogeneity. In fact, our negative-stain EM analysis indicated that the orientation of the bound NZ-1 Fab relative to the target PDZ domains was variable. In particular, *Aa*RseP (181-PA14-184) complexed with the NZ-1 Fab showed a high degree of conformational flexibility between the two molecules. Crystallographic analysis showed that only the junction residues of the target PDZ-N domain made direct contacts with the NZ-1 Fab in the 181-PA14-184 mutant. In addition, MD simulations indicated that the intermolecular interaction between Glu1′ in PA14 and NZ-1 can readily be broken in this mutant. For the 181-PA14-184 mutant, we attempted to optimize the insertion site by deleting the turn residues to eliminate the intermolecular space between the target PDZ-N domain and the bound NZ-1 Fab, as this insertion site protruded from the globular domain. Nevertheless, it seems that some conformational flexibility still exists (Fig. 7 ▸ *b*) due to the absence of specific interactions between the NZ-1 Fab and the target PDZ-N domain other than through the inserted PA14 tag. In contrast, the orientation of the NZ-1 Fab seemed to be relatively fixed in the complex with *Aa*RseP (235-PA14-236). Lys255 in PDZ-C interacted with Asp72 in the NZ-1 light chain in complex 1 of the crystal structure of the PDZ tandem (235-PA14-236) with the NZ-1 Fab, and this interaction was reproduced in the MD simulation. We therefore hypothesized that it could stabilize the subunit arrangements in the complex. However, the structural alignment suggested that only the PA14 residues were involved in intermolecular interactions with the NZ-1 Fab because the interaction mediated by Lys255 was broken in the atomic models that fit the 3D reconstruction from the EM analysis (Supplementary Figs. S5*b*, S8*b* and S8*d*). As the PA14 tag was inserted into a loop that packs against a flat surface on this insertion mutant, the orientation of the NZ-1 Fab in the EM model might be locked in position by steric hindrance from the flat surface of the PDZ domain. These observations indicated that creating an NZ-1-labeled complex with reduced flexibility will require further optimization of the insertion strategy by considering both specific interactions and steric hindrance between the target and the Fab.

In conclusion, we have optimized the insertion method of the NZ-1 epitope to reduce structural changes in the target protein. Using a target with known structure, we showed that inserting a PA14 tag is more suitable for NZ-1 labeling compared with PA12 insertion. The advantage of the PA14 tag is that it can reduce structural changes in the target protein during labeling and structure determination even when it is inserted into sterically hindered sites such as loops that pack against the protein. If these advantages are general properties of PA14, the use of PA14 as an inserted epitope tag will expand the range of application of the NZ-1 labeling technique. To further advance our generalizable method of antibody-assisted structural analysis for application in EM studies, future work will optimize the insertion site to fix the orientation of the NZ-1 Fab relative to the target and minimize conformational variability. Other optimization challenges, such as Fab dissociation upon vitrification, will also need to be addressed. This endeavor may require mutations of both the epitope and the paratope. Computational approaches, such as the modeling of PA14-inserted mutants and/or NZ-1 Fab mutants followed by MD simulation to assess complex rigidity, will aid these future optimizations. Ultimately, a generalizable method for antibody labeling that produces stable and rigid antibody–target complexes will be an important new tool for high-resolution structural analysis by both X-ray crystallo­graphy and cryo-EM.

## Supplementary Material

PDB reference: NZ-1 Fab–PDZ tandem complex, mediated by epitope inserted into the PDZ-N domain, 7cqc

PDB reference: mediated by epitope inserted into the PDZ-C domain, 7cqd

Supplementary Table and Figures. DOI: 10.1107/S2059798321002527/ji5021sup1.pdf

## Figures and Tables

**Table 1:** Data-collection statistics for Fab complexes Values in parentheses are for the highest resolution shell.

PDZ tandem	181-PA14-184	235-PA14-236
Space group	*P*2_1_2_1_2_1_	*P*2_1_
*a*, *b*, *c* (Å)	52.36, 75.20, 172.53	81.36, 80.18, 168.54
α, β, γ (°)	90, 90, 90	90, 95.7, 90
No. of complexes in asymmetric unit	1	2
X-ray source	BL-5A, PF	BL-17A, PF
Wavelength (Å)	1.0000	0.9800
Resolution limits (Å)	45.68–2.50 (2.60–2.50)	38.99–3.20 (3.36–3.20)
No. of unique reflections	24452 (2685)	35827 (4745)
Completeness (%)	99.9 (99.6)	99.5 (99.7)
Multiplicity	6.6 (6.8)	3.4 (3.5)
〈*I*/σ(*I*)〉	13.1 (1.5)	8.0 (1.2)
*R*_merge_	0.093 (1.469)	0.115 (0.965)
*R*_meas_	0.102 (1.589)	0.137 (1.141)
CC_1/2_	0.998 (0.674)	0.994 (0.774)

**Table 2:** Refinement statistics for Fab complexes Values in parentheses are for the highest resolution shell.

PDZ tandem	181-PA14-184	235-PA14-236
Resolution limits (Å)	42.97–2.50 (2.55–2.50)	38.99–3.20 (3.25–3.20)
*R*_work_ †	0.230 (0.363)	0.265 (0.415)
*R*_free_ ‡	0.258 (0.457)	0.286 (0.431)
No. of non-H atoms		
Total	4740	7901
Complex 1		
PDZ-N	801	—
PDZ-C	651	734
NZ-1 Fab (H)	1597	1575
NZ-1 Fab (L)	1641	1641
Complex 2		
PDZ-N	—	—
PDZ-C	—	734
NZ-1 Fab (H)	—	1576
NZ-1 Fab (L)	—	1641
Solvent	50	0
Average *B* factors (Å^2^)		
Overall	72.80	121.44
Complex 1		
PDZ-N	61.26	—
PDZ-C	122.09	132.18
NZ-1 Fab (H)	63.70	121.75
NZ-1 Fab (L)	68.26	121.11
Complex 2		
PDZ-N	—	—
PDZ-C	—	146.29
NZ-1 Fab (H)	—	117.34
NZ-1 Fab (L)	—	109.51
Solvent	55.91	—
R.m.s.d. from ideality		
Bond lengths (Å)	0.002	0.003
Bond angles (°)	0.54	0.85
Ramachandran plot		
Favored (%)	95.17	94.81
Outliers (%)	0.33	0.29
PDB code	7cqc	7cqd

† *R*_work_ is the crystallographic *R* factor calculated for the working set consisting of 95% of reflections used in refinement.

‡ *R*_free_ is the crystallographic *R* factor calculated for the test set consisting of 5% of reflections excluded from refinement.
