# Supplementary material for: Moving toward generalizable NZ-1 labeling for 3D structure determination with optimized epitope-tag insertion
Source: Acta Crystallogr D Struct Biol. 2021 Apr 19;77(Pt 5):645–62. doi: 10.1107/S2059798321002527 (PMC8098476; doi:10.1107/S2059798321002527)
Supplement: Supplementary file 1 [file d-77-00645-sup1.pdf]

Acta Cryst D

STRUCTURAL BIOLOGY

Volume 77 (2021)

Supporting information for article:

Moving toward generalizable NZ-1 labeling for 3D structure determination with optimized epitope-tag insertion

Risako Tamura-Sakaguchi, Rie Aruga, Mika Hirose, Toru Ekimoto, Takuya Miyake, Yohei Hizukuri, Rika Oi, Mika K. Kaneko, Yukinari Kato, Yoshinori Akiyama, Mitsunori Ikeguchi, Kenji Iwasaki and Terukazu Nogi

Supplementary Information

Supplementary Table S1. Plasmid list

Plasmid	Vector	Encoded protein	Reference or source
pGEX-2T			Cytiva (GE healthcare)
pNO1499	pGEX-2T	PDZ tandem	(Hizukuri *et al.*, 2014)
pNY1468	pGEX-2T	PDZ tandem (235-PA14-236)	This study
pNY1493	pGEX-2T	PDZ tandem (181-PA14-184)	This study
pET-11c			Agilent Technologies (Stratagene)
pUC118			Takara Bio
pTWV228			Takara Bio
pYH124	pTWV228	HA-MBP-RseA(LY1)148	(Hizukuri & Akiyama, 2012)
pKK11	pTWV228	*Ec*RseP-His_6-Myc	(Kanehara *et al.*, 2001)
pKK34	pTWV228	*Ec*RseP(E23Q)-His_6-Myc	(Kanehara *et al.*, 2001)
pNO1457	pUC118	*Aa*RseP-His_8	This study
pNO1461	pUC118	*Aa*RseP(E18Q)-His_8	This study
pNY1478	pUC118	*Aa*RseP(235-PA14-236)-His_8	This study
pNY1498	pUC118	*Aa*RseP(181-PA14-184)-His_8	This study
pTM748	pTWV228	*Aa*RseP-His_8	This study
pTM749	pTWV228	*Aa*RseP(E18Q)-His_8	This study
pTM750	pTWV228	*Aa*RseP(235-PA14-236)-His_8	This study
pTM751	pTWV228	*Aa*RseP(181-PA14-184)-His_8	This study

Supplementary Figures

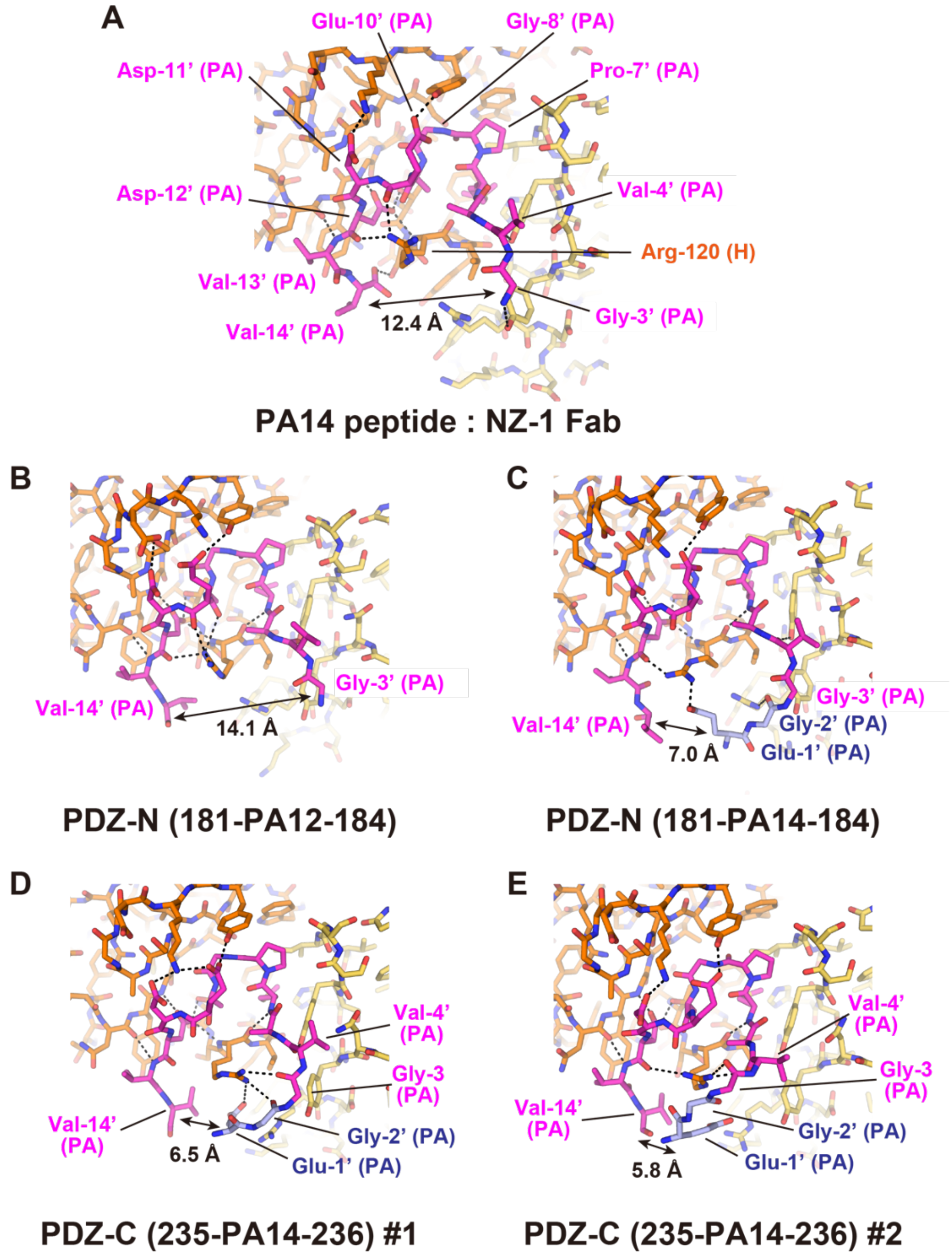

Supplementary Figure S1

Conformations of the PA14 or PA12 tags in the crystal structures of NZ-1 Fab complexes. (A) PA14 peptide (Protein Data Bank accession code: 4YO0) (B) PA12-

inserted PDZ-N domain (181-PA12-184 mutant; Protein Data Bank accession code: 6AL1) (C) PA14-inserted PDZ-N domain (181-PA14-184 mutant; this study) (D) PA14-inserted PDZ-C domain (235-PA14-236 mutant; this study) in complex #1 (E) PA14-inserted PDZ-C domain (235-PA14-236 mutant; this study) in complex #2. The heavy and light chains of the NZ-1 Fab are colored dark and light orange, respectively. The PA12 tag and the C-terminal 12 residues of the PA14 tag are colored in magenta while the two N-terminal residues (Glu-1'and Gly-2') of the PA14 tag are colored light blue. The Cα-Cα distances between Glu-1' and Val-14' in PA14 and between Gly-3' and Val-14' in PA12 are each indicated with an arrow. The four N-terminal PA14 residues (Glu-1' to Val-4') from PDZ-C (235-PA14-236) adopted a different conformation in complex #2 as compared to those observed in the other complexes.

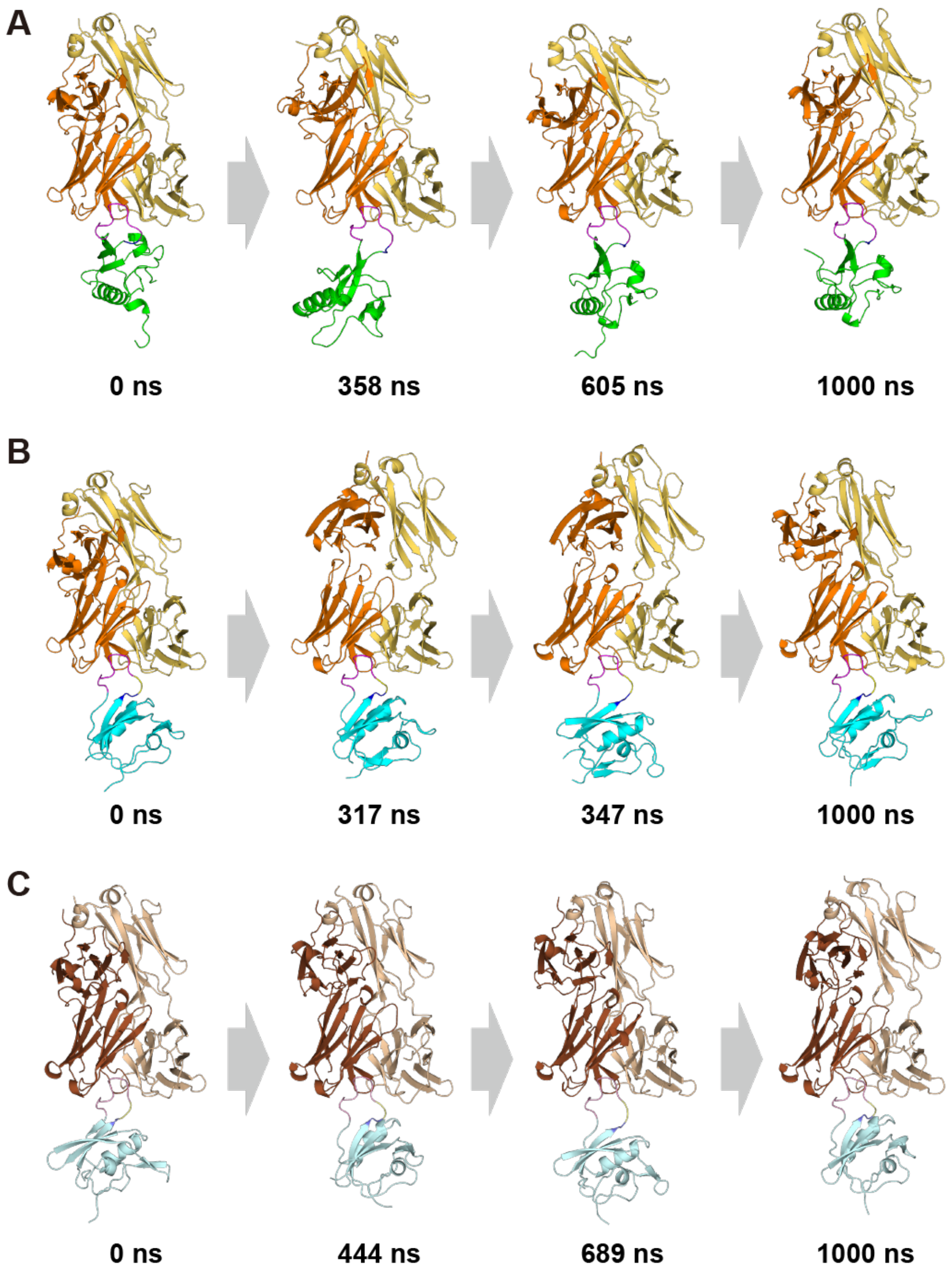

Supplementary Figure S2

Snapshot models from the MD trajectories. (A) The trajectory for PDZ-N (181-PA14-184) complexed with the NZ-1 Fab. Snapshots taken during each 1-μs simulation are shown as ribbon models. The heavy and light chain of the NZ-1 Fab are colored dark and light orange, respectively. The PDZ-N domain (residues 113-206) is colored green. The

inserted PA14 tag is shown in magenta with Glu-1' and Gly-2' colored blue for emphasis. The snapshot at 0 ns shows the energy-minimized initial model of PDZ-N (181-PA14-184) complexed with the NZ-1 Fab while the 1000 ns snapshot shows the final model from the 1-μs simulation. The snapshot at 358 ns showed the highest RMSD relative to the initial model. The snapshot at 605 ns was the representative model selected by PCA in Supplementary Fig. 5A. (B) The trajectory initialized from the energy-minimized model of PDZ-C (235-PA14-236) complexed with the NZ-1 Fab from complex #1. The NZ-1 Fab is colored as in panel (A). The PDZ-C domain (207-292) is colored cyan. For the inserted PA14 tag, Glu-1' and Gly-2' are colored blue while Gly-3' and Val-4' are colored yellow. The remaining 10 residues of the PA14 tag are colored magenta. The snapshot at 317 ns was the representative model selected from the PCA in Supplementary Fig. 5B. The snapshot at 347 ns was the model showing both the low RMSD relative to the PDZ-C model in complex #2 and the long distance between Lys-255 on PDZ-C and Asp-72 on the NZ-1 light chain in Supplementary Fig. 5C. (C) The trajectory initialized from the energy-minimized model of PDZ-C (235-PA14-236) complexed with the NZ-1 Fab from complex #2. The heavy and light chain of the NZ-1 Fab are colored dark and light brown, respectively. For the inserted PA14 tag, Glu-1' and Gly-2' are colored light blue while Gly-3' and Val-4' are colored light yellow. The remaining 10 residues of PA14 are colored light magenta. The snapshot at 444 ns was the representative model selected by PCA in Supplementary Fig. 5B. The snapshot at 689 ns was a model showing both a low RMSD relative to the PDZ-C model in complex #2 and a long distance between Lys-255 on PDZ-C and Asp-72 on the NZ-1 light chain in Supplementary Fig. 5C.

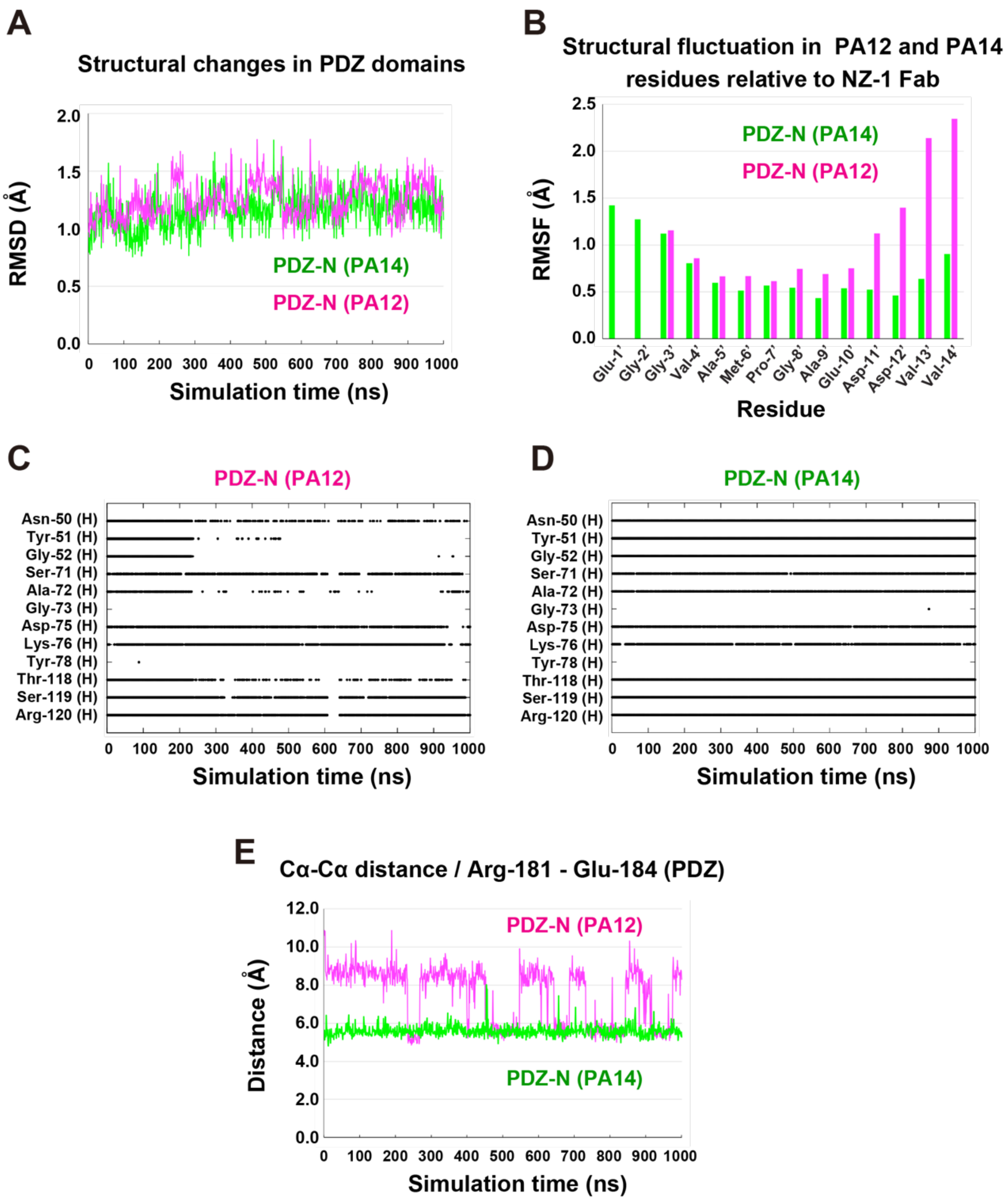

Supplementary Figure S3

MD simulations for PDZ-N (181-PA12-184) complexed with the NZ-1 Fab. (A) Trajectories for the target PDZ-N structure. RMSDs were calculated for the snapshot models relative to the initial model to estimate the structural changes in the PDZ-N

domain during the simulation trajectories. The inserted PA12 residues and the flexible N-terminal region upstream of the PDZ-N domain were excluded from the RMSD calculation. RMSDs plotted for trajectories of the PDZ-N domain are shown in magenta. (B) Structural fluctuations in PA12 tag residues. In the RMSF calculations, the models of the complex from the trajectory were aligned to the initial model based on the V_H region of the NZ-1 Fab, and the structural fluctuation from the averaged structure was calculated for each residue on PA14. (C) Residues on the NZ-1 Fab located within 4 Å of Asp-11' or Asp-12' on PA12. The plots indicate if each residue contacts Asp-11' or Asp-12' in each snapshot on the trajectory. Asp-11' and Asp-12' were separated from Tyr-51 and Gly-52 on the heavy chain after 240 sec. The contacts with Ala-72 and Thr-118 on the heavy chain were also broken frequently during the simulation. (D) Residues on the NZ-1 Fab located within 4 Å of Asp-11' or Asp-12' on PA14. In contrast to PA12, almost all the listed residues except for Gly-73 and Tyr-78 on the heavy chain made stable contacts with Asp-11' or Asp-12' during the simulation, indicating that PA14 stayed inside the antigen-binding pocket. (E) Fluctuations during MD simulations for the Cα-Cα distance between Arg-181 and Glu-184. Arg-181 and Glu-184 are the junction residues connected to the PA12 tag. The Cα-Cα distances were plotted for the trajectories with PDZ-N (181-PA12-184) and PDZ-N(181-PA14-184) in magenta and green, respectively.

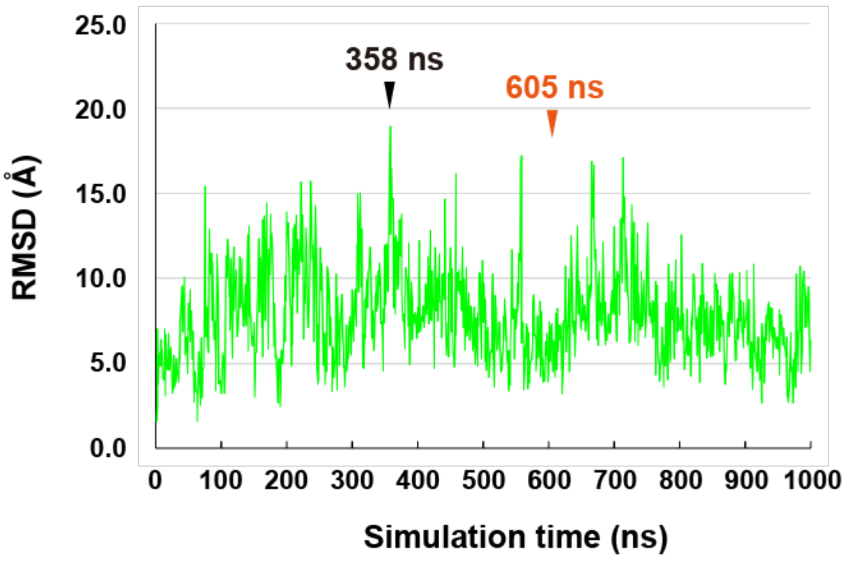

Supplementary Figure S4

Deviation of the position of the PDZ-N domains relative to the NZ-1 Fab in the MD simulations. The snapshot models were aligned based on the V_H region to the initial model, which was prepared by energy minimization of the crystal structure of PDZ-N (181-PA14-184) complexed with the NZ-1 Fab. Subsequently, the RMSDs for the PDZ-N domains were calculated between the initial model and the respective snapshots on the trajectory. PDZ-N (181-PA14-184) showed the highest RMSD value relative to the initial model at 358 ns. The snapshot at 605 ns was selected as the representative model from the PCA in Supplementary Fig. 5A.

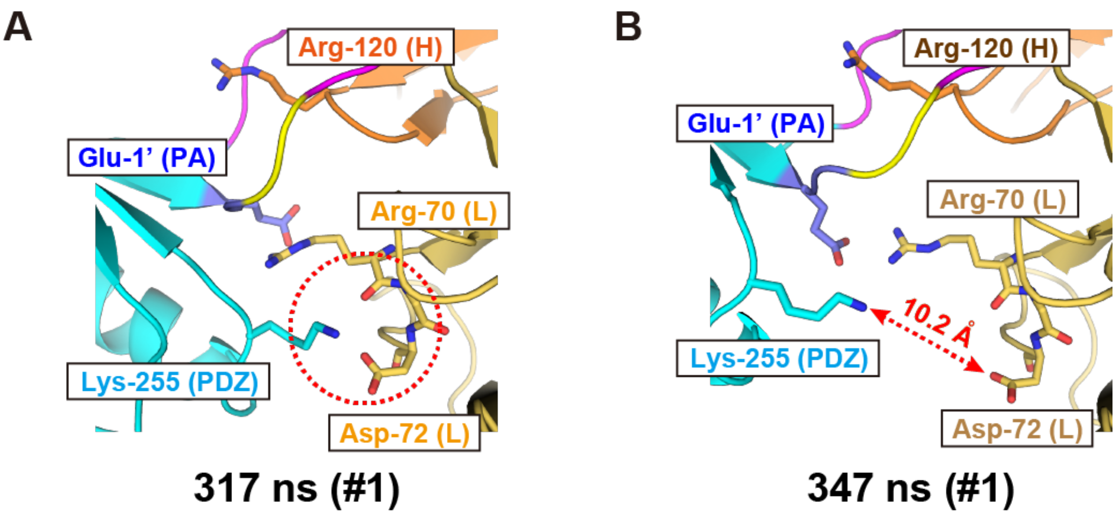

Supplementary Figure S5

Binding interfaces of the snapshot models of PDZ-C (235-PA14-236) complexed with the NZ-1 Fab. (A) Residues at the binding interface in the 317-ns snapshot from the trajectory initialized from complex #1. Lys-255 on the PDZ-C domain formed hydrogen bonds with the side chain of Asp-72 and the main chain of Arg-70 on the NZ-1 light chain. These interactions seemed to draw the PDZ-C domain close to the NZ-1 Fab. (B) Residues at the binding interface in the 347-ns snapshot from the trajectory initialized from complex #1. The side chain amino group of Lys-255 on the PDZ-C domain was separated by 10.2 Å from the side chain carboxyl group of Asp-72 on the NZ-1 light chain.

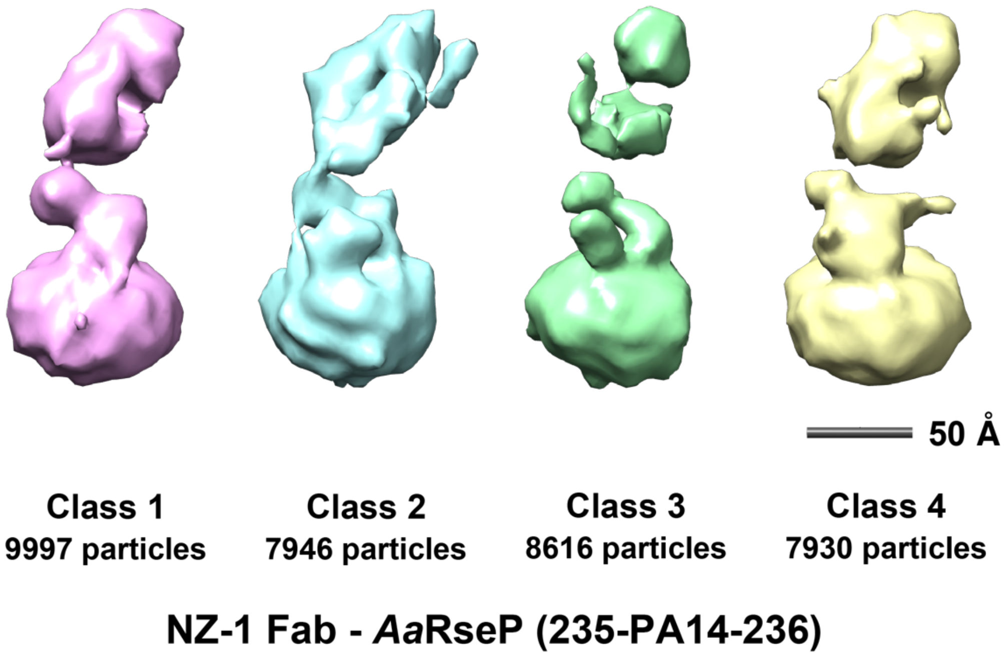

Supplementary Figure S6

3D reconstruction models of *Aa*RseP (235-PA14-236) complexed with the NZ-1 Fab from negative-stain EM. Four different 3D models reconstructed from the 2D class averages are shown in different colors. Class 1 corresponds to the 3D model shown in Fig. 7C. The 3D models of classes 2 to 4 were aligned onto that of class 1 based on the position of the putative NZ-1 Fab domain. The models are displayed at lower threshold levels than that in Fig. 7C. The numbers of particles used for the reconstruction are indicated below each model.

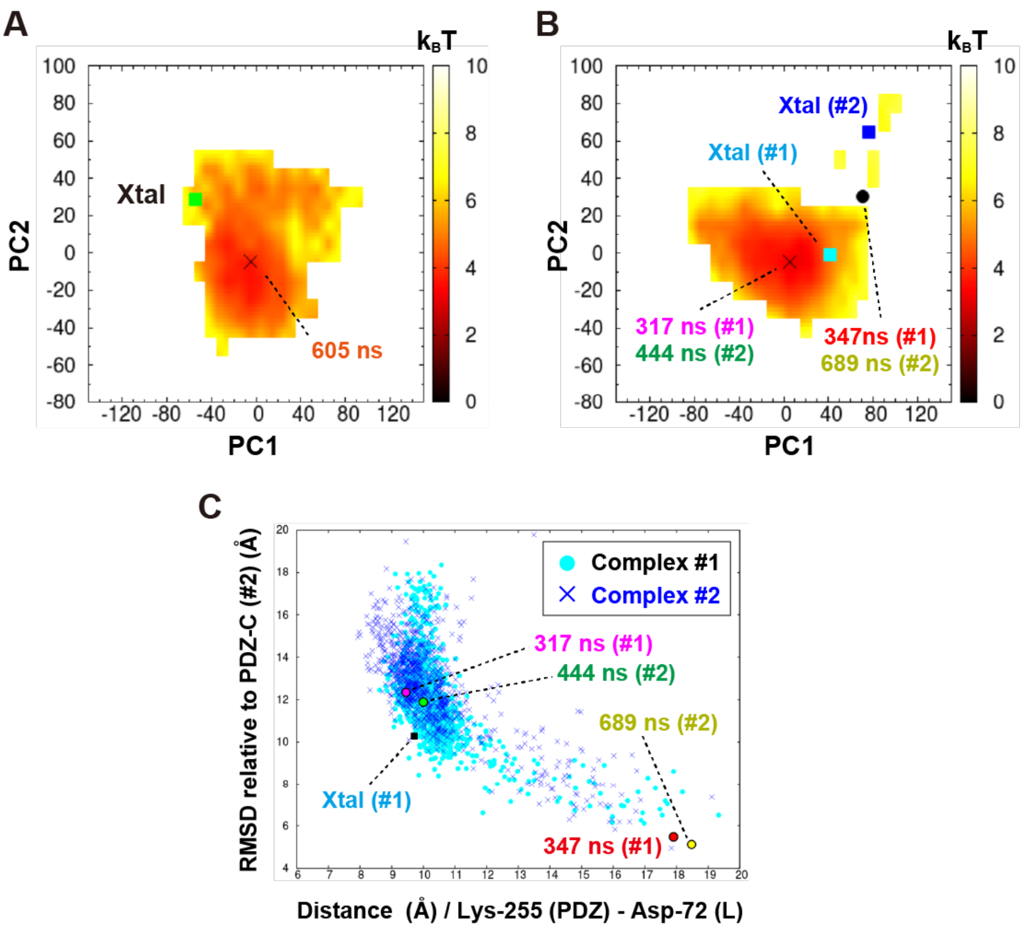

Supplementary Figure S7

Analysis of structural fluctuation and domain orientation within the Fab-PDZ complex. (A) Quantification of the structural fluctuation in PDZ-N (181-PA14-184) complexed with NZ-1 Fab. PCA was performed for the snapshots on the trajectory to quantify the structural fluctuation. The structural distribution is represented as a 2D normalized histogram of -ln(Z) plotted on a PC map with a pixel size of 10 Å × 10 Å, where Z was the probability of a given conformation. The values for the free energy are given in units of k_BT (color bar), where k_B is the Boltzmann constant and T is the temperature. The initial energy minimized model of PDZ-N complexed with the NZ-1 Fab (Xtal), which was almost identical to the crystal structure, is indicated by the green square on the plot. The 605-ns snapshot was selected from the cluster with the maximum probability on the

PC map. (B) Quantification of structural fluctuation of PDZ-C (235-PA14-236) complexed with the NZ-1 Fab. Snapshots from two trajectories initialized from each of complexes #1 and #2 were merged and subjected to the PCA. The structural distribution is represented as a 2D normalized histogram, as in panel (A). The initial energy minimized models of the NZ-1 Fab-PDZ-C pair constructed from complex #1 and #2, which are almost identical to the corresponding crystal structures, are indicated by a cyan (Xtal (#1)) or a blue (Xtal (#2)) squares on the plot. The 317-ns snapshot from complex #1 (magenta) and the 444-ns snapshot from complex #2 (green) were selected from the cluster with the maximum probability on the PC map. The 347-ns snapshot from complex #1 (red) and 689-ns snapshot from complex #2 (yellow), which showed both a low RMSD relative to the PDZ-C model in complex #2 and a long distance between Lys-255 on PDZ-C and Asp-72 on the NZ-1 light chain, are also plotted on the PC map. (C) Analysis of domain orientation within the Fab-PDZ complex based on comparison to complex #2. The RMSD relative to the PDZ-C model in complex #2 for each snapshot was plotted against the distance between the Cα atoms of Lys-255 on PDZ-C and Asp-72 on the NZ-1 light chain. The snapshots from the trajectories for complexes #1 and #2 are indicated with cyan circles and blue cross marks, respectively. In complex #2, the PDZ-C domain was separated from the NZ-1 Fab while the hydrogen bond between Lys-255 and Asp-72 was broken. Therefore, the both a low RMSD and the long distance between Lys-255 and Asp-72 indicate higher conformational similarity to complex #2. The 317-ns snapshot from complex #1 (magenta) and the 444-ns snapshot from complex #2 (green) were located in proximity to the majority of snapshots on the PC map, whereas the 347-ns snapshot from complex #1 (red) and 689-ns snapshot from complex #2 (yellow) appeared in a minor population of outliers.

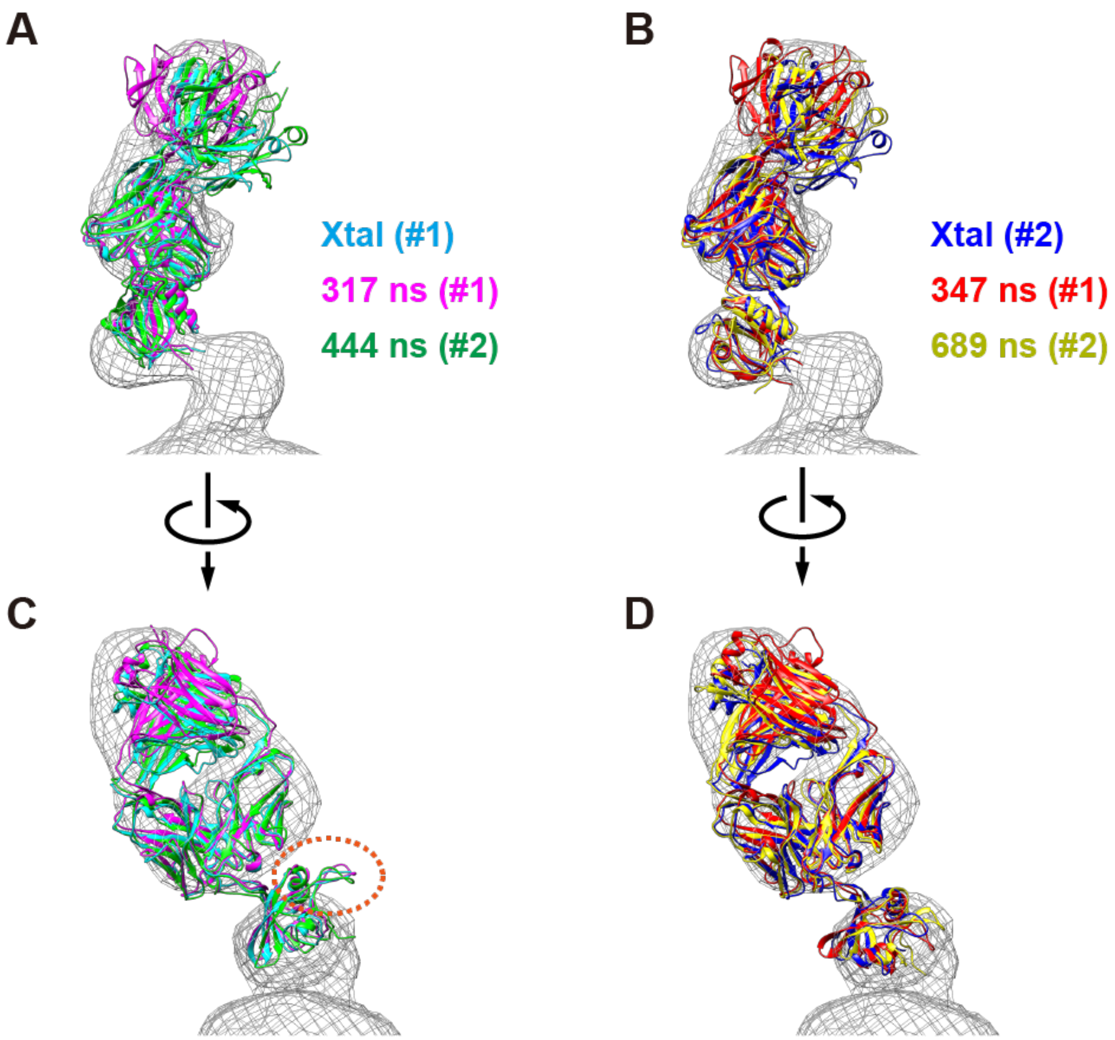

Supplementary Figure S8

Structural alignment of the NZ-1 Fab-PDZ-C complexes onto the 3D reconstruction model. (A) Complex #1 (cyan) from the crystal structure of the PDZ tandem (235-PA14-236) complexed with the NZ-1 Fab, the 317-ns snapshot from the MD trajectory initialized from complex #1 (magenta), and the 444-ns snapshot from the trajectory initialized from complex #2 (green) were all aligned onto the 3D reconstruction model of *Aa*RseP (235-PA14-236) complexed with the NZ-1 Fab. (B) Complex #2 (blue), the 347-ns snapshot from the trajectory initialized from complex #1 (red), and the 689-ns snapshot from the trajectory initialized from complex #2 (yellow) were aligned onto the 3D

reconstruction model. Panels (C) and (D) are side-views of panels (A) and (B), respectively. For the three complexes shown in (A) and (C), alignment of the models based on the position of the Fv region placed the βA-βB loops of the PDZ-C models outside the EM map, as highlighted with red dotted circle, while the same loops on the three models in (B) and (D) largely fit within a lobe of density in the EM map.
